# Direct and indirect hyperpolarisation of amines using *para*hydrogen†
†Electronic supplementary information (ESI) available: Full experimental procedures, characterisation data and example spectra. See DOI: 10.1039/c8sc00526e


**DOI:** 10.1039/c8sc00526e

**Published:** 2018-03-09

**Authors:** Wissam Iali, Peter J. Rayner, Adel Alshehri, A. Jonathan. Holmes, Amy J. Ruddlesden, Simon B. Duckett

**Affiliations:** a Centre for Hyperpolarisation in Magnetic Resonance (CHyM) , Department of Chemistry , University of York , Heslington , YO10 5DD , UK . Email: simon.duckett@york.ac.uk

## Abstract

Nuclear Magnetic Resonance (NMR) and Magnetic Resonance Imaging (MRI) are two widely used techniques for the study of molecules and materials. Hyperpolarisation methods, such as Signal Amplification By Reversible Exchange (SABRE), turn typically weak magnetic resonance responses into strong signals. In this article we detail how it is possible to hyperpolarise the ^1^H, ^13^C and ^15^N nuclei of a range of amines. This involved showing how primary amines form stable but labile complexes of the type [Ir(H)_2_(IMes)(amine)_3_]Cl that allow *para*hydrogen to relay its latent polarisation into the amine. By optimising the temperature and *para*hydrogen pressure a 1000-fold per proton NH signal gain for deuterated benzylamine is achieved at 9.4 T. Additionally, we show that sterically hindered and electron poor amines that bind poorly to iridium can be hyperpolarised by either employing a co-ligand for complex stabilisation, or harnessing the fact that it is possible to exchange hyperpolarised protons between amines in a mixture, through the recently reported SABRE-RELAY method. These chemical refinements have significant potential to extend the classes of agent that can be hyperpolarised by readily accessible *para*hydrogen.

## Abstract 2

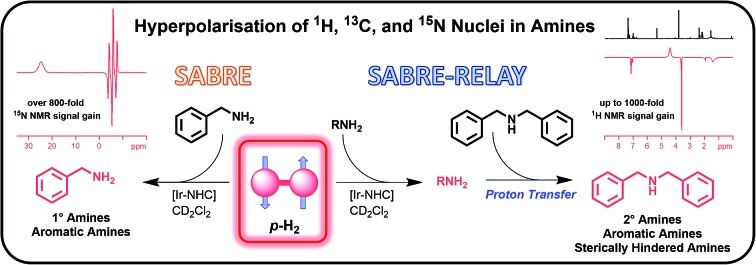
Para-hydrogen achieves the hyperpolarisation of amines *via* SABRE.

## Introduction

**Scheme 1:**
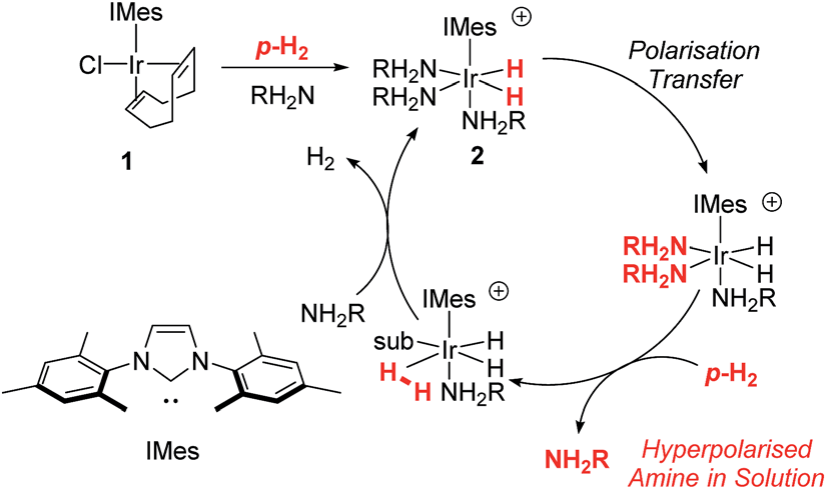
Route to SABRE hyperpolarisation of an amine, NH_2_R.

Hyperpolarisation methods are used to overcome the inherent insensitivity of Nuclear Magnetic Resonance (NMR) spectroscopy and Magnetic Resonance Imaging (MRI) where their use may lead to dramatic time and cost savings. One such hyperpolarisation method, *Para*hydrogen Induced Polarisation (PHIP),^1^ produces the required non-Boltzmann nuclear spin distribution by the incorporation of *para*hydrogen (*p*-H_2_), an example of a nuclear singlet, into a suitable substrate molecule. This effect was shown to yield an enhanced NMR signal in 1987 (ref. 2) and has been the subject of intense investigation.^1^,^3^–^6^ A drawback of PHIP though, is the requirement for chemical change, caused by *p*-H_2_ addition to an unsaturated centre such as an alkene. However, recently a *p*-H_2_ technique that does not change the chemical identity of the sensitised molecule, called Signal Amplification By Reversible Exchange (SABRE), was reported.^7^,^8^ In this process, *p*-H_2_ is not directly incorporated into the substrate. Instead, polarisation is transferred *via* the *J*-coupling network that exists within a metal complex that co-locates *p*-H_2_ derived hydride ligands and a weakly bound substrate (ligand).^9^–^11^ Ligand exchange with excess unbound substrate and *p*-H_2_ enables the build-up of a pool of polarised substrate molecules in solution in a catalytic fashion as shown in Scheme 1.^12^ The SABRE polarisation of ^1^H nuclei typically utilises a ^4^*J*_HH_ coupling between the catalysts hydride and substrate ligand protons. Tessari *et al.* have quantified these small spin–spin couplings to be ≈1.2 Hz.^13^ Alternatively, stronger ^2^*J*_HN_ couplings have now been used to achieve ^15^N polarisation transfer at micro-Tesla fields in a variant known as SABRE-SHEATH (SABRE-in shield enables alignment transfer to heteronuclei).^14^,^15^ Intra-molecular spin–spin coupling networks within the substrate subsequently enables transfer to remote spins which do not exhibit direct coupling to the hydride ligands.^16^

One of the most effective precatalysts for this process is [IrCl(COD)(IMes)] (**1**) [where IMes = 1,3-bis(2,4,6-trimethylphenyl)imidazol-2-ylidene, COD = *cis*,*cis*-1,5-cyclooctadiene] which, after reaction with H_2_ and an excess of substrate, typically forms [Ir(H)_2_(IMes)(substrate)_3_]Cl in protic solvents such as methanol.^17^ Neutral active catalysts of the type [Ir(H)_2_(Cl)(IMes)(substrate)_2_] have also been reported to achieve similar results.^18^ These metal based polarisation transfer catalysts have been shown to act on a range of substrates that contain multiple bonds to nitrogen, such as nicotinamide,^19^,^20^ isoniazid,^21^,^22^ metronidazole,^23^ pyrazole,^24^ imines,^25^ diazirines^26^ and nitriles,^27^ and lead to polarised ^1^H, ^13^C, ^15^N, ^19^F, ^29^Si, ^31^P, and ^129^Sn nuclei that yield substantially enhanced NMR responses in just a few seconds.^19^,^28^–^33^ In fact, ^1^H polarisations of 50% have been reported, while for ^15^N, values of over 20% have been achieved.^19^,^23^

While SABRE-induced polarisation can also be achieved using in-field rf. transfer methods,^34^–^37^ whose efficiency varies with pulse sequence,^37^–^39^ spontaneous polarisation transfer occurs readily at low-field and it is this method we employ here. Moreover, as predicted,^9^ it has also been established that SABRE can be used to produce hyperpolarised singlet states^40^ with long-lifetimes through transfer in ultra-low field, or after the implementation of rf. transfer.^41^–^46^ Hence the diversity of applications found for this approach is growing and it clearly reflects not only a successful medium to test hyperpolarisation concepts but a potential route to transform the analytical potential of NMR.^47^–^50^

In this article, we introduce a new class of substrate into the SABRE repertoire, the amine. This is achieved by the formation of iridium–amine complexes of type [Ir(H)_2_(IMes)(RNH_2_)_3_]Cl (**2**, Scheme 1), whose kinetic behaviour is determined. Whilst the synthesis and use of iridium–amine complexes has been reported for catalytic transformations such as hydrogenation,^51^–^53^ we use them here for polarisation transfer catalysis. We have recently shown a limited number of amines are amenable to SABRE.^54^ Here, we start by detailing the hyperpolarisation of ammonia and benzylamine (BnNH_2_) and its associated optimisation to achieve large NMR signal enhancements. We then show how hyperpolarisation can be achieved in a range of primary amines. Upon changing to sterically bulky primary amines, secondary amines or aromatic amines, we show that an active SABRE catalyst does not form upon reaction with **1**. However, we exemplify co-ligand and relayed polarisation transfer protocols to overcome this limitation and hence expand further the range of amines amenable to polarisation by *p*-H_2_.

## Results and discussion

### Direct ^1^H hyperpolarisation of ammonia and BnNH_2_ by SABRE

Our objective was to investigate the efficiency of the SABRE polarization of amines and ammonia and to determine their ligand exchange dynamics. A 5 mM solution of **1** in dry dichloromethane-*d*_2_ containing an ≈6-fold excess of NH_3_ relative to **1** at 298 K was therefore prepared. The aprotic solvent ensures that we maintain the necessary *J*-coupling network in [Ir(H)_2_(IMes)(NH_3_)_3_]Cl (**2-NH_3_**) during the study, as rapid ^2^H exchange results to form ND_3_ in deuterated protic solvents. This complex yields a hydride signal at *δ* –23.8, alongside a broad response at *δ* 0.47 for free NH_3_. The corresponding equatorial and axial NH_3_ ligand ^1^H NMR signals of **2-NH_3_** appear at *δ* 2.19 and 2.88 respectively. 2D ^1^H–^15^N HMQC measurements were subsequently used to locate the corresponding ^15^N signals for these ligands at *d*_axial_ –47.8 and *d*_equ_ –35.5. Full characterisation data for **2-NH_3_** is available in the ESI.† 54 EXSY methods were then used to probe NH_3_ and H_2_ loss in **2-NH_3_**. At 298 K, the associated rate constant for NH_3_ loss proved to be 1.64 s^–1^ while that of H_2_ loss is 0.32 s^–1^. For comparison, the dissociation rate for pyridine in [Ir(H)_2_(IMes)(py)_3_]Cl is 13.2 s^–1^ and suggests a higher stability for **2-NH_3_** which agrees with the greater basicity of NH_3_ relative to pyridine.^55^

**Fig. 1:**
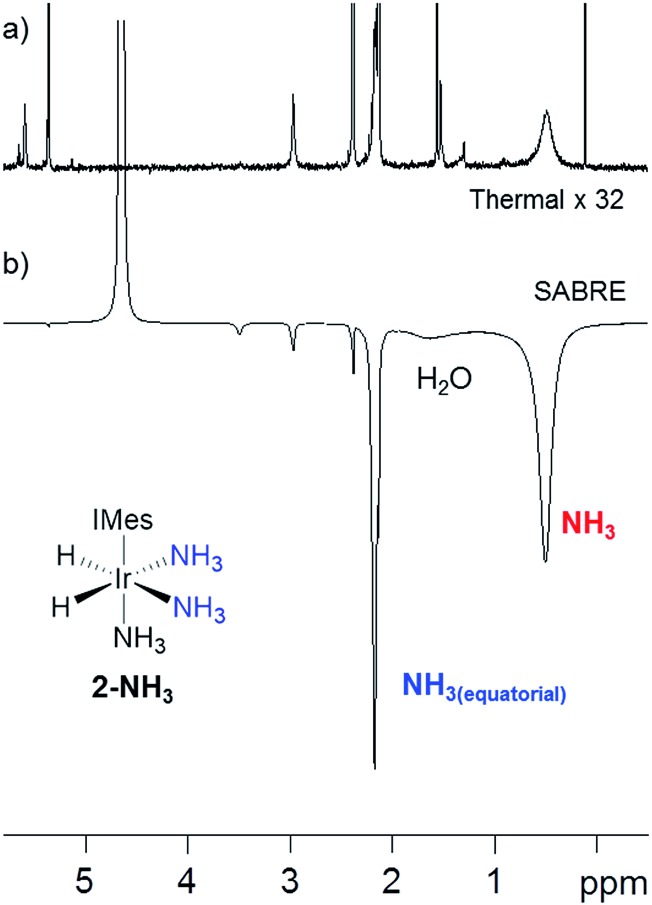
(a) The thermally polarised ^1^H NMR SABRE spectrum (× 32 vertical expansion) recorded of **2-NH_3_** (formed by reaction of **1** with NH_3_ and H_2_) in dichloromethane-*d*_2_ at 298 K. (b) The corresponding SABRE polarised 9.4 T ^1^H NMR spectrum after transfer under *p*-H_2_ at 60 G. The hyperpolarised responses of free 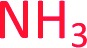 and 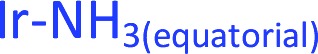 of **2-NH_3_** and residual H_2_O are indicated.

As **2-NH_3_** undergoes both NH_3_ and H_2_ loss in solution, we sought to prove that it underwent SABRE catalysis. Thus, a 3 bar pressure of *p*-H_2_ was introduced at 298 K and polarisation transfer was conducted at 60 G. A ^1^H NMR spectrum at 9.4 T was then recorded which showed a 154-fold signal enhancement per proton for the free NH_3_ response while the corresponding equatorial ligand signal, at *δ* 2.19, showed a 77-fold enhanced response (Fig. 1). Hence **2-NH_3_** acts as a SABRE catalyst as it produces a hyperpolarised free ammonia response. In the presence of water, the observed signal enhancement of the protons in free NH_3_ decreased to 40-fold per proton, matching that now observed for the equatorially bound NH_3_ ligand This drop is reflected in the signal at *δ* 1.88, for what is a H_2_O response, exhibiting a 75-fold signal gain per proton due to concomitant proton exchange; the ratio of **2-NH_3_** : H_2_O : NH_3_ in this sample was 1 : 5 : 17.5. Under these conditions, the *T*_1_ value for free NH_3_ in the presence of the active SABRE catalyst was measured by inversion recovery to be 5.5 s.

The SABRE-induced hyperpolarisation of benzylamine (BnNH_2_) was also investigated. A sample containing **1** (5 mM) and BnNH_2_ (10 eq.) in dichloromethane-*d*_2_ solution was exposed to 3 bar of H_2_. The immediate formation of [Ir(H)_2_(IMes)(BnNH_2_)_3_]Cl (**2-BnNH_2_**) was observed. It gives a characteristic hydride resonance in the ^1^H NMR spectrum at *δ* –23.97. Full characterisation data for this product is available in the ESI.† Interestingly, the ^1^H NMR spectrum of **2-BnNH_2_** showed that the BnNH_2_ ligand that lies *trans* to hydride, yields inequivalent responses for its NH_2_ protons at *δ* 4.92 and 2.30, and CH_2_ protons at *δ* 3.60 and 3.18. This is due to hindered rotation around the Ir–N bond which results in an up/down distinction for the resonances of the equatorial ligand. In contrast, the axial ligand yields single responses which are equivalent at *δ* 4.24 (NH_2_) and *δ* 3.83 (CH_2_) due to free rotation on the NMR timescale about the Ir–N bond. The corresponding EXSY-derived rate constant for equatorial BnNH_2_ loss from **2-BnNH_2_** was 3.33 s^–1^ while the rate of H_2_ loss was 2.83 s^–1^ at 298 K. Hence the rate of BnNH_2_ loss is higher than that of NH_3_ loss in **2-NH_3_**. This difference is due to NH_3_ forming a stronger Ir–N bond as reflected in their relative *p*K_b_ values and suggests that it might perform better under SABRE that NH_3_.

**Fig. 2:**
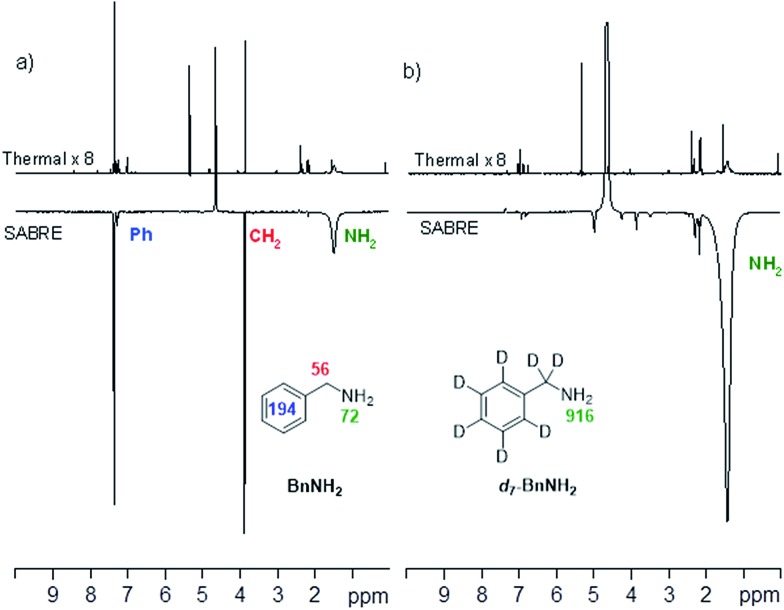
(a) ^1^H NMR spectra of BnNH_2_, thermally polarised, top, and hyperpolarised, bottom. (b) ^1^H NMR spectra for *d*_7_-BnNH_2_, thermally polarised, top, and hyperpolarised, bottom.

This was examined by *p*-H_2_-based polarisation transfer at 60 G which resulted in hyperpolarised free BnNH_2_ in solution. The signal enhancements were quantified to be 72-(NH_2_), 56-(CH_2_) and 194-fold (Ph) per proton as shown in Fig. 2a. However, by using *d*_7_-BnNH_2_ instead we were able to focus the SABRE polarisation into the two amino protons alone and this led to an improved signal enhancement of 916-fold per proton (Fig. 2b).

In order to investigate the *T*_1_ contribution to this effect we determined values for BnNH_2_ and *d*_7_-BnNH_2_ at 9.4 T. BnNH_2_ proved to have effective *T*_1_ values of 1.1 s (NH_2_) and 4.7 s (CH_2_) respectively while its ^2^H-labelled variant exhibited a similar 1.1 s *T*_1_ value for the amino group in the presence of the active catalyst. Hence, the improved NH signal gain seen with *d*_7_-BnNH_2_ is due to a reduction in spin dilution which leads to more efficient SABRE transfer. The relaxation rates for BnNH_2_ and *d*_7_-BnNH_2_ are both slower in the absence of the active SABRE catalyst in agreement with earlier reports that the catalyst plays a role in reducing relaxation times due to reversible binding. Consequently, BnNH_2_ now shows *T*_1_ values of 9.0 s (NH_2_) and 11.0 s (CH_2_), whereas *d*_7_-BnNH_2_ has a *T*_1_ value of 10.1 s for its NH_2_ group.

### Effect of catalyst to substrate ratio on SABRE polarisation

Previous studies have shown that the SABRE effect is dependent upon the catalyst to substrate ratio as a consequence of kinetic and relaxation effects.^19^,^55^ Therefore, we studied the effect of changing the ratio of BnNH_2_ relative to **1** from 4-fold to 20-fold in a series of further experiments, undertaking the associated SABRE transfer studies at 60 G and 298 K. It was found that similar total polarisation levels result within experimental error during these experiments (see ESI†). Hence, we conclude that the observed signal enhancements under these conditions are essentially independent of ligand excess which suggests that slow exchange and fast relaxation within the catalyst restrict the maximum polarisation level.

### Effect of *p*-H_2_ pressure on SABRE polarisation of BnNH_2_

As SABRE derives its polarisation from *p*-H_2_, it could be the limiting reagent in this catalytic process and therefore affect the observed substrate polarisation level.^19^ Up until this point, we have been utilising 3 bar pressure of *p*-H_2_ which reflects an *ca.* 6-fold excess when compared to the 50 mM substrate present in a 5 mm NMR tube. A sample containing **1** (5 mM), BnNH_2_ (50 mM, 10 eq.) in dichloromethane-*d*_2_ solution was therefore prepared and exposed to between 2 and 4 bar of *p*-H_2_. The resulting signal gains, after polarisation transfer at 60 G, are shown in Fig. S14 (see ESI†) and a strong dependence on *p*-H_2_ pressure is seen. This is consistent with the fact that H_2_ exchange takes place after ligand dissociation and the remaining equatorially bound BnNH_2_ ligand will experience a higher level of latent *p*-H_2_ polarisation (see Scheme 1). When *d*_7_-BnNH_2_ is examined with 4 bar of *p*-H_2_, the NH signal gain increases to 1079-fold per proton from the 916-fold signal gain achieved with 3 bar.

### Effect of temperature on SABRE polarisation of BnNH_2_

The temperature at which SABRE is conducted is also known to affect the efficiency of the polarisation transfer due to changes in the lifetime of the SABRE-active catalyst. We found here that cooling a dichloromethane-*d*_2_ solution containing **1**, BnNH_2_ and 3 bar *p*-H_2_ to 288 K results in a reduction in the level of signal enhancement when compared to 298 K data (Fig. S15, ESI†). Conversely 308 K gave an improved response with the overall polarisation level increasing by ∼40%. This fits with the observed rate constant for BnNH_2_ dissociation increasing to 9.85 s^–1^ from the 3.33 s^–1^ value at 298 K. We therefore conclude the retained polarisation level in BnNH_2_ is improved by the faster rate of substrate dissociation and shorter catalyst lifetime. For NH_3_, a 251-fold ^1^H signal gain per NH proton is observed at 308 K when compared to the 154-fold value at 298 K. This is consistent with the increase in the NH_3_ dissociation rate constant to 10.42 s^–1^ at 308 K when compared to 1.64 s^–1^ at 298 K.

### SABRE transfer to ^13^C and ^15^N

**Fig. 3:**
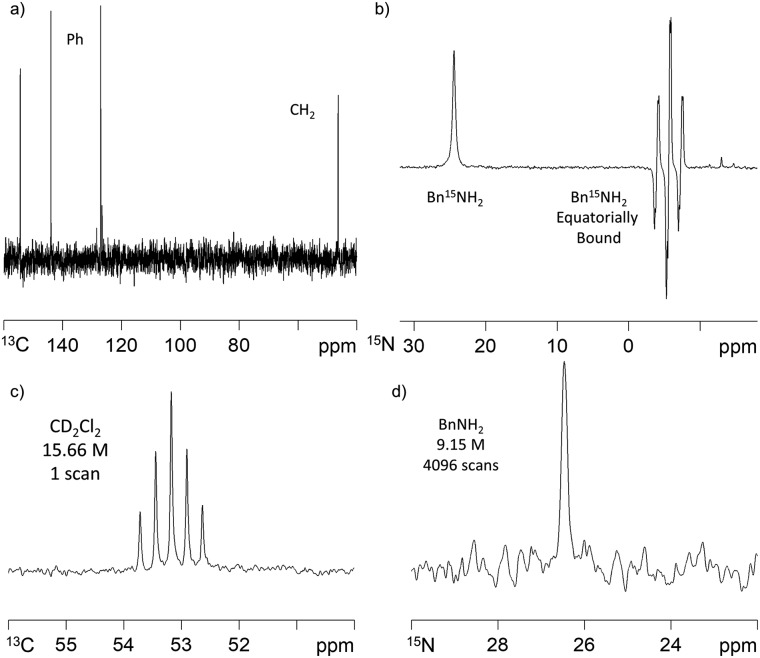
(a) ^1^H–^13^C refocussed INEPT NMR spectrum of hyperpolarised BnNH_2_ (35 mM) achieved *via***2-BnNH_2_** (5 mM) under SABRE in dichloromethane-*d*_2_ solution after transfer at 60 G and 308 K; (b) ^15^N NMR spectrum of ^15^N-labelled Bn^15^NH_2_ (35 mM) after SABRE transfer *via***2-BnNH_2_** (5 mM) at 60 G and 308 K which gives rise to hyperpolarised resonances for free (*δ* 24.42) and equatorially bound (*δ* –5.59) substrate; (c) single scan thermally polarised ^13^C NMR spectrum in CD_2_Cl_2_ (15.66 M) and (d) 4096 scan thermally polarised ^15^N NMR spectrum of BnNH_2_ (9.15 M).

SABRE-induced hyperpolarisation of ^13^C was also observed for BnNH_2_. Whilst polarisation transfer into the *ortho* phenyl carbon was readily observed using a standard ^13^C acquisition sequence after polarisation transfer 60 G under 4 bar *p*-H_2_, the other ^13^C resonances had poor signal-to-noise ratios. We overcame this by using a ^1^H–^13^C refocussed INEPT experiment that gave rise to a spectrum showing all 5 carbon environments after polarisation transfer at 60 G. We utilised long-range *J*-H–C-couplings to transfer this polarisation. ^13^C signal gains of up to 65-fold were achieved using this method (Fig. 3a). We further note that there is a very strong polarisation transfer field dependence on the BnNH_2_^13^C signal intensities which is consistent with earlier reports on pyridine.^30^

When Bn^15^NH_2_ is used instead of BnNH_2_, the detection of a hyperpolarised ^15^N response is readily evident as shown in Fig. 3b. The ^15^N signal gain for the free material in solution proved to be ∼880-fold after polarisation transfer at 60 G and 308 K. The equatorially bound ^15^N resonance at *δ* –5.59, is 4 times larger than the free amine signal. As the ratio of free amine to equatorially bound Bn^15^NH_2_ in solution is actually 7 : 2, the rate of Bn^15^NH_2_ loss must be relatively slow, even at 308 K. Under this 60 G condition, polarisation transfer is likely to occur *via* the ^3^*J*_HH_ coupling between the Bn^15^NH_2_ and the hydride ligands. To investigate the effect of using a ^2^*J*_HN_ coupling we repeated this measurement after polarization transfer within a μ-metal shield (*ca.* 350-fold shielding). Under these SABRE-SHEATH type conditions,^14^,^15^ an ∼800-fold ^15^N-signal gain was observed and further optimisation may therefore be needed to maximise this response. The corresponding ^1^H signal gains with this ^15^N labelled material after transfer at 60 G were now 33-(NH_2_), 34-(CH_2_) and 52-fold (Ph). These compare to the analogous values of 72-, 56- and 192-fold respectively with Bn^14^NH_2_. Interestingly, the ^1^H polarisation levels therefore decrease with ^15^N addition and we propose that this is an example of spin dilution.

### Expanding the substrate range

**Fig. 4:**
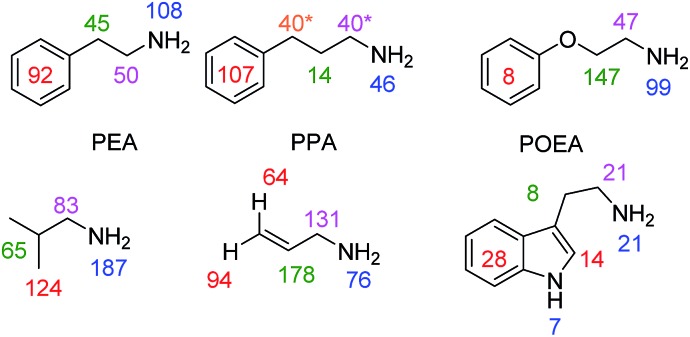
Amine substrates polarised by SABRE using precatalyst **1** in dichloromethane-*d*_2_ solution. Per proton signal gains are given for the indicated ^1^H sites (* average across two sites due to peak overlap) observed at 9.4 T. Corresponding ^1^H NMR spectra for thermally polarised and SABRE polarised experiments are given in the ESI.†

In order to test the generality of amine polarisation *via* SABRE, we prepared a series of samples containing **1** (5 mM) and 10 eq. of the substrates shown in Fig. 4 in dichloromethane-*d*_2_ solution. These substrates include a number of primary amines and each is successfully hyperpolarised after transfer at 60 G upon reaction with **1** and *p*-H_2_. In fact, SABRE polarisation of phenylethylamine (PEA) and phenylpropylamine (PPA) results in strong signal enhancements and transfer is found to proceed across the corresponding C_2_ and C_3_ carbon chains into their phenyl rings. For PEA we found that the NH_2_^1^H signal gain is actually increased to 108-fold per proton compared to the 72-fold BnNH_2_ value, and that the CH_2_CH_2_ bridge gave 50-fold (NCH_2_) and 45-fold (CH_2_) enhancements per proton. The 5-proton containing phenyl group gave a 92-fold gain per proton.

Spin-isolation of the phenyl group, by introducing an ether linkage, as in phenoxyethylamine (POEA) resulted in signal enhancements of 99-(NH_2_), 47-(NCH_2_), 147-(CH_2_O) and as expected, just 8-fold (Ph) per proton for our test sample. We therefore conclude that polarisation transfer across the oxygen linker is inefficient at 60 G and a stronger aliphatic proton response results. The amines isobutylamine, allylamine and tryptamine were also studied as shown in Fig. 4. In all cases, the formation of [Ir(H)_2_(IMes)(amine)_3_]Cl was indicated (see ESI†) and polarisation transfer results.

When secondary amines, such as dibenzylamine, were examined, no evidence for the formation of an active SABRE catalyst was observed. A similar result was observed for sterically hindered primary amines, such as isopropylamine and aromatic amines, such as aniline. Sterically demanding substrates, such as 2,6-lutidine, have been previously shown to be unable to be polarised using SABRE.^56^ A full list of the amines probed in this study is available in the ESI.† We therefore postulate that sterically demanding or electron deficient amines fail to activate and form the necessary [Ir(H)_2_(IMes)(amine)_3_]Cl SABRE catalyst.

**Fig. 5:**
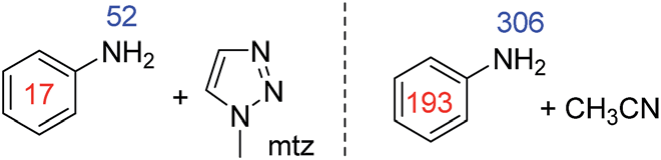
^1^H NMR signal gains per proton observed for the indicated aniline resonances when hyperpolarised by SABRE in the presence of the described co-ligand at 9.4 T.

This problem could be overcome for aniline by the addition of the co-ligand 1-methyl 1,2,3-triazole (mtz) or CH_3_CN. For the corresponding sample containing **1** (5 mM), aniline (10 eq.) and mtz (3 eq.) in dichloromethane-*d*_2_ we achieved signal enhancements of 51-fold for the NH_2_ group and 17-fold for the phenyl group, per proton. These signal gains are summarised in Fig. 5. When CH_3_CN (8 eq.) is used instead of mtz, the polarisation levels increase to 306-(NH_2_) and 193-fold (Ph) per proton. The active complex in this SABRE process was characterised as [Ir(H)_2_(IMes)(aniline)_2_(CH_3_CN)]Cl and yields a distinctive hydride resonance at *δ* –24.78 (see ESI†). Utilisation of such a co-substrate strategy was however unsuccessful for the secondary amines as detailed in the ESI.†

### Indirect hyperpolarisation of amines by SABRE-RELAY

**Scheme 2:**
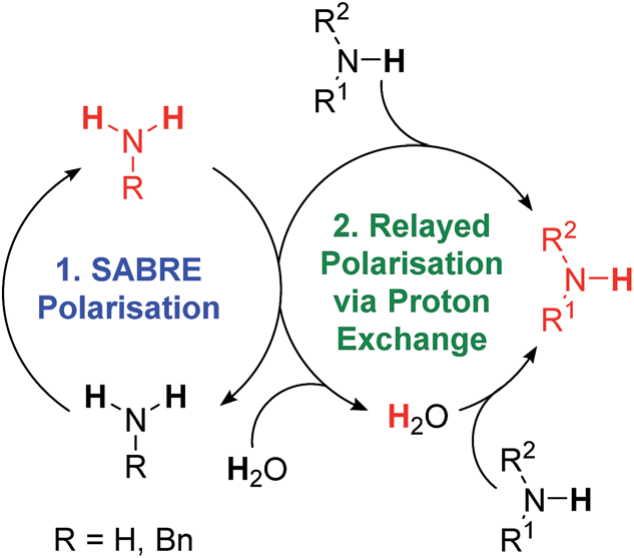
SABRE-RELAY polarisation of amines. (1) SABRE polarisation of an intermediary transfer agent, in this case a primary amine or ammonia. (2) Polarisation is then relayed into the target amine *via* proton exchange, either directly or *via* residual water present in the sample.

As expected, substrate binding to the metal centre is needed for polarisation transfer to occur. We hypothesised that these amines might also be hyperpolarised indirectly. In this scenario, hyperpolarisation of a primary amine or ammonia is achieved and subsequent proton exchange, which may be mediated by residual water, allows for a polarised proton to be shuttled into the non-SABRE-active amine. Subsequent intra-substrate polarisation transfer then relays the signal gain more widely in this agent (Scheme 2).

**Fig. 6:**
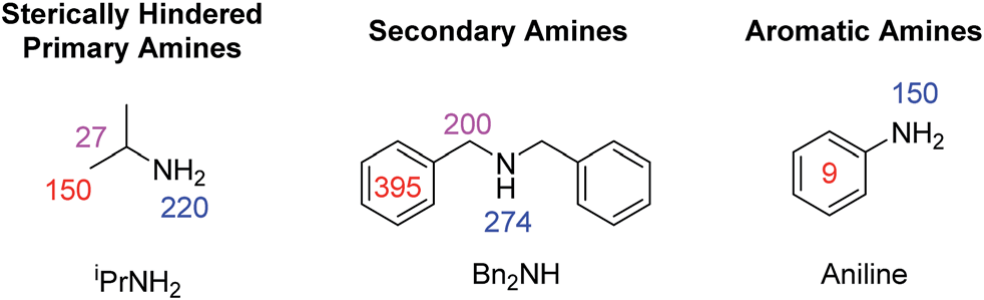
^1^H NMR signal gains observed per proton for the indicated amine resonances when hyperpolarised by SABRE-RELAY using **2-NH_3_** at 9.4 T.

In order to test this hypothesis, a series of samples containing **1** (5 mM), target amine (10 eq.) and NH_3_ (3–5 eq.) were prepared in dichloromethane-*d*_2_ solution. **2-NH_3_** formed in all cases as confirmed by the presence of a hydride resonance in the corresponding ^1^H NMR spectra at *δ* –23.8. Polarisation transfer was then conducted at 60 G, and the resulting signal gains that were observed at 9.4 T are presented in Fig. 6.

For isopropylamine (^i^PrNH_2_), the SABRE-RELAY polarised NH_2_ signal showed a 220-fold signal gain while 27- and 150-fold enhancements were seen for the CH and CH_3_ resonances respectively. This reflects a breakthrough as ^i^PrNH_2_ was unable to be directly polarised by SABRE due to its steric bulk preventing adequate binding. Dibenzylamine (Bn_2_NH) was also successfully polarised using this method, and yields ^1^H signal gains of 274-(NH), 200-(CH_2_) and 395-fold (Ph) per proton. Additionally, a ^13^C spectrum can be acquired in a single scan on these materials after polarisation transfer at 60 G such that a 475-fold signal gain for the CH_2_ resonance is observed. Full NMR spectra are available in the ESI.† Furthermore, the aromatic amine, aniline, now exhibits a 150-fold NH_2_ proton signal enhancement and a 9-fold signal gain for the phenyl ring under analogous conditions. We note that these signal gains are lower than those seen when CH_3_CN is used as a co-ligand to achieve direct SABRE transfer as detailed in Fig. 5. We suggest that this difference in behaviour arises because a 60 G polarisation transfer field is no-longer optimal for intra-molecular polarisation transfer after proton exchange. This is clearly is not the case for transfer *via* directly bound aniline and the complexes scalar coupling network which is in fact commonly maximised for ^1^H transfer at 60 G.

From these results we can conclude that the SABRE-RELAY effect is able to polarise sterically hindered primary amines, secondary amines and aromatic amines that are not themselves accessible to SABRE. Thus, the scope of amine polarisation is vastly increased.

## Conclusions

In summary, we have shown here how SABRE can be used to hyperpolarise a series of primary amines. This substrate extension opens up the SABRE approach to operate with a much wider range of analytes than was previously thought possible, as we extend beyond the original aromatic N-heterocycles, imines and nitriles. Activity is achieved by the formation of a series of complexes of the form [Ir(H)_2_(IMes)(amine)_3_]Cl. Relaxation studies, in conjunction with ligand dissociation rate measurements were used to demonstrate that the high relative stability of these complexes acts to limit the degree of SABRE signal gain. This hypothesis is consistent with the fact that increasing the *p*-H_2_ pressure or reaction temperature leads to improved signal gains. Therefore, significant catalyst optimisation will be important if very high levels of hyperpolarisation are to be achieved by this route in the future.

Nonetheless, in the case of BnNH_2_, ^1^H NMR signal enhancement values of ∼100-fold per NH proton were achieved for benzylamine using [IrCl(COD)(IMes)]. Consequently, when *d*_7_-benzylamine was used, the resulting focusing of the hyperpolarisation into the NH_2_ resonance resulted in a 900-fold signal enhancement per proton at 9.4 T with a *p*-H_2_ pressure of 3 bar. This value reduced to 33-fold for Bn^15^NH_2_ after transfer at 60 G. Hence, we predict that further improvements can be made through a more detailed study of the effect of isotopic labelling.^18^,^19^,^57^ We have also demonstrated transfer to ^13^C and ^15^N with diagnostic NMR spectra being collected at a 35 mM concentration in a single scan. We predict that application of high-field SABRE transfer techniques,^34^–^37^,^39^ such as the LIGHT-SABRE^38^ approach, might subsequently enable this process to work inside the magnet, but note that a rigorous study of the effect the polarisation transfer field plays on the resulting signal enhancement levels is justified.

In the course of these studies we found that sterically hindered primary amines, secondary amines and aromatic amines were unable to form an active SABRE catalyst of the type [Ir(H)_2_(IMes)(amine)_3_]Cl. This meant that direct polarisation transfer *via* such a complex was not possible. We found for aniline that the addition of a co-ligand such as CH_3_CN overcame this problem *via* the formation of [Ir(H)_2_(IMes)(aniline)_2_(CH_3_CN)]Cl such that signal enhancements of up to 306-fold per NH proton could be achieved.

An indirect route was described to overcome this limitation more generally, such that hindered primary amines, secondary amines and aromatic amines can be hyperpolarised by SABRE-RELAY.^54^ Now, a SABRE-hyperpolarised intermediary, such as ammonia, is able to readily transfer polarisation into agents such as isopropylamine, benzylamine and aniline *via* either direct proton exchange or mediated by residual water present in the sample. This approach expands the range of amines that can be hyperpolarised without changing their chemical identity through interactions with *p*-H_2_.

Given the increase in signal intensity that is observed for the amines in this study, we are now working towards their use as agents for mechanistic study^58^–^64^ in transfer hydrogenation,^65^,^66^ hydroamination,^67^,^68^ and vitally important N_2_ fixation reactions.^69^–^71^ Additionally, since phenylethylamine is a naturally occurring monoamine based alkaloid that acts as a promoter of catecholamine (dopamine and norepinephrine) release in plants and animals we expect these observations to be of wide interest.^72^,^73^ Furthermore, the SABRE-RELAY method^54^ has recently been shown to offer a route to hyperpolarise an even larger range of hydrogen transfer acceptors using OH functional groups. Optimisation of the intermediaries NH polarisation level reflects a key part to optimisation of this technique and hence these results will be of interest to any potential developer.

## Conflicts of interest

The authors declare no conflicts of interest.

## Supplementary Material

Supplementary information
