# Supplementary material for: Direct and indirect hyperpolarisation of amines using parahydrogen
Source: Chem Sci. 2018 Mar 9;9(15):3677–84. doi: 10.1039/c8sc00526e (PMC5935062; doi:10.1039/c8sc00526e)
Supplement: Supplementary file 1 [file SC-009-C8SC00526E-s001.pdf]

Electronic Supplementary Material (ESI) for Chemical Science.
This journal is © The Royal Society of Chemistry 2018

Supporting Information

Direct and Indirect Hyperpolarisation of Amines using *para*Hydrogen

Wissam Iali,[a] Peter J. Rayner,[a] Adel Alshehri,[a] A. Jonathan. Holmes,[a] Amy J. Ruddlesden[a] and Simon B. Duckett*[a]

[a] Centre for Hyperpolarisation in Magnetic Resonance, Department of Chemistry, University of York, Heslington, YO10 5DD

Contents

1 NMR polarisation transfer experiment data
- **1.1 SABRE polarisation transfer method**
- **1.2 SABRE-RELAY polarisation transfer method with NH_3**
- **1.3 Polarisation factors**
- **1.4 NMR Spectrometer**
- **1.5 Pulse Sequences**

2 Representative SABRE NMR spectra and enhancement factors

3 Representative SABRE NMR spectra and enhancement factors using a co-ligand

4 Representative SABRE-RELAY NMR spectra and enhancement factors

5 Optimisation of the SABRE polarisation of $BnNH_2$
- **5.1 Effect of varying the pressure of *p*-H_2**
- **5.2 Effect of temperature of SABRE catalysis**

6 Ligand exchange dynamics of 2-NH_3 and 2-$BnNH_2$

7 NMR Characterization data for the active SABRE catalysts
- **7.1 2-NH_3**
- **7.2 2-$BnNH_2$**
- **7.3 2-PEA**
- **7.4 [Ir(H_2)(IMes)(aniline)$_2$(MeCN)]Cl**

1 NMR polarisation transfer experiment data

1.1 SABRE polarisation transfer method

The polarisation transfer experiments that are reported in this study were conducted in 5 mm NMR tubes that were equipped with a J. Young's tap. Samples for these polarisation transfer experiments were based on 5 mM solutions of [IrCl(COD)(IMes)] and the indicated substrate and loadings in methanol-d_4 or dichloromethane-d_2 (0.6 mL). The samples were degassed prior to the introduction of NH_3. Subsequently, *para*hydrogen (p-H_2) at a pressure of ca. 3 bar was added. Samples were then shaken for 10 s in the specified fringe field of an NMR spectrometer (9.4 T) before being rapidly transported into the magnet for subsequent interrogation by NMR spectroscopy. This whole process takes ca. 15 seconds to achieve.

1.2 SABRE-RELAY polarisation transfer method with NH_3

The polarisation transfer experiments that are reported were conducted in similar 5 mm NMR tubes equipped with J. Young's valves. Samples were based on 5 mM solutions of [IrCl(COD)(IMes)], NH_3 (5-10 eq.), and the indicated additional substrate at the specified loading in dichloromethane-d_2 (0.6 mL). The samples were degassed prior to the introduction of p-H_2 at a pressure of ca. 3 bar. They were shaken for 10 s in the specified fringe field of an NMR spectrometer (9.4 T) before being rapidly transported into the magnet for subsequent interrogation by NMR spectroscopy.

1.3 Polarisation factors

For calculation of the ^1H and ^{13}C signal enhancements the following formula was used:

$$E = \frac{SI(pol)}{SI(unpol)}$$

Where, E = enhancement level, SI(pol) = signal of polarised sample, SI(unpol) = signal of unpolarised (reference) sample. Experimentally, both spectra were recorded on the same sample using identical acquisition parameters, including the receiver gain. The raw integrals of the relevant resonances in the polarised and unpolarised spectra were then used to determine the enhancement levels. The quoted values reflect the signal strength gain (fold) per proton/phosphorus nucleus in the specified group.

^{13}C enhancements were calculated by taking the raw integral of the $^{13}CD_2Cl_2$ peak observed from the solvent in the sample after equilibration inside the magnet for 1 minute. CD_2Cl_2 was present in each sample at a concentration of 15.67 M and the resulting SABRE-Relay

hyperpolarised signal was then scaled according to the concentration of substrate in solution (given in section 2) to give the final enhancement value for ^{13}C. These values were validated by further measurements on samples containing $CHCl_3$ as an internal standard.

1.4 NMR Spectrometer

Spectra were typically acquired on a 400 MHz Bruker, Avance III console using a 5 mm BBI probe which was tuned to ^{1}H, ^{13}C, ^{31}P or ^{15}N as specified. Resonances are referenced relative to the residual proton signal of the indicated deuterated solvent.

1.5 Pulse Sequences

- **Refocused INEPT**

  INEPT based measurements of ^{13}C and ^{15}N NMR spectra were recorded using a standard refocused INEPT experiment with decoupling during acquisition as outlined in Fig. S1. The evolution delay (d4) is optimised to 1/2 x J_{XH} and obtains in-phase X-magnetisation through the addition of a refocusing delay (d3) which is set to 1/(6 x J_{XH}). Values of J_{XH} are given in section 2.

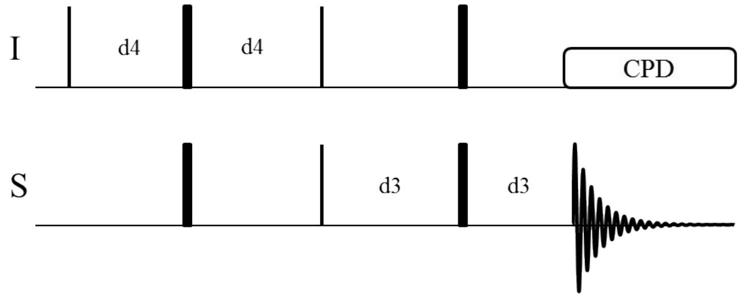

Fig. S1: Ineptrd pulse sequence utilised for SABRE-RELAY derived heteronuclei detection.

2 Representative SABRE NMR spectra and enhancement factors

- **Ammonia**

Fig. S2 reflects a series of single scan ^{1}H NMR spectra employing ammonia in CD_2Cl_2: a) ^{1}H NMR spectrum thermally polarised x16 vertical scale, b) ^{1}H SABRE NMR spectrum hyperpolarisation; using the conditions: [IrCl(COD)(IMes)] (5 mM), CD_2Cl_2 (0.6 mL), NH_3; ^{1}H enhancement (fold/per proton): 94-(NH_3), x1 vertical scale.

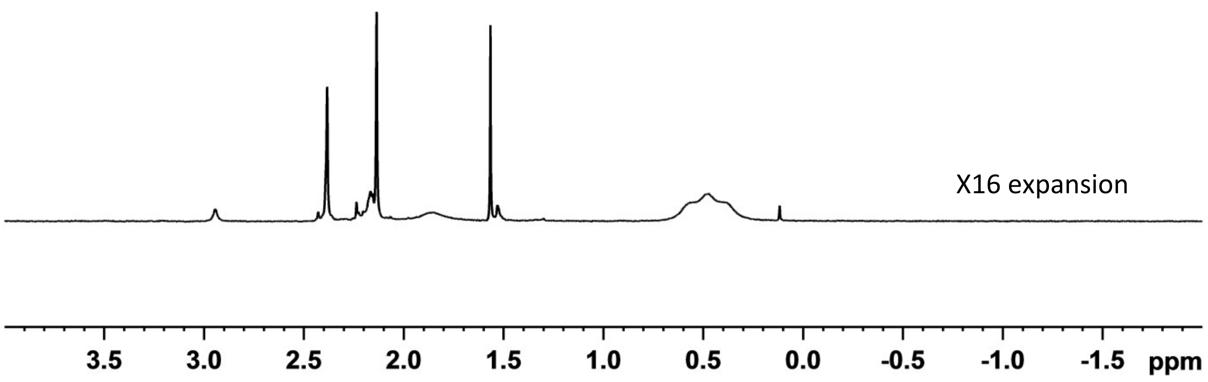

Fig. S2a Thermally polarised ^{1}H NMR reference spectrum for ammonia in CD_2Cl_2

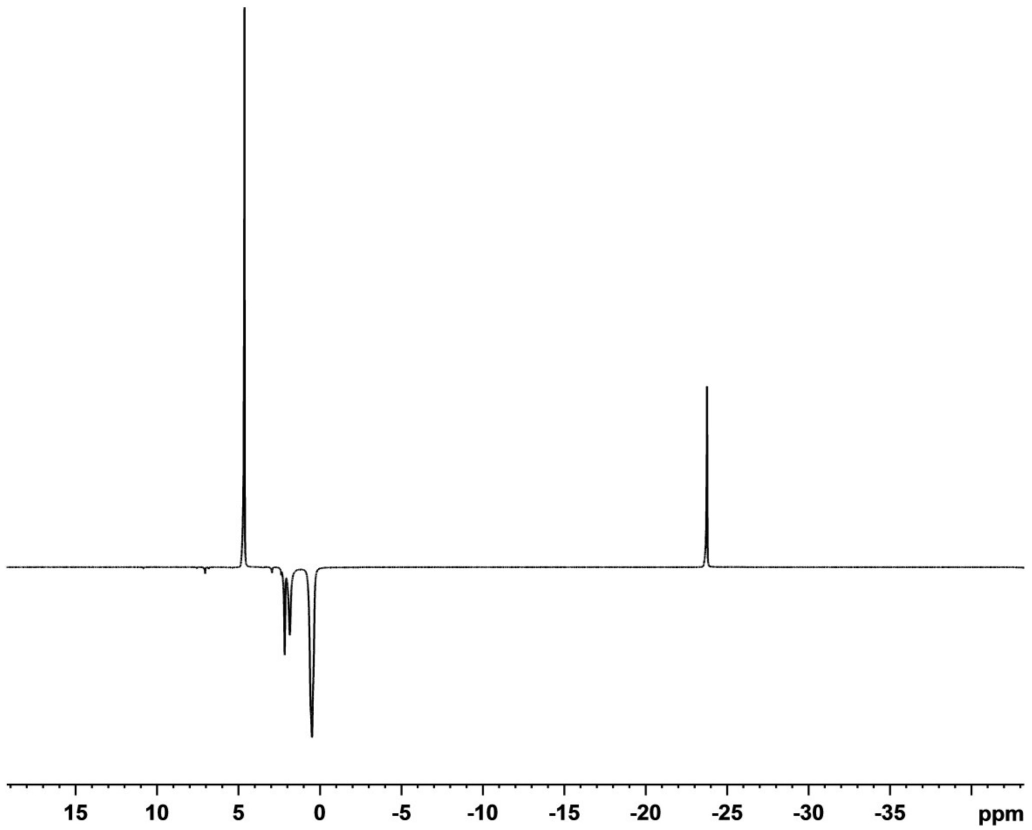

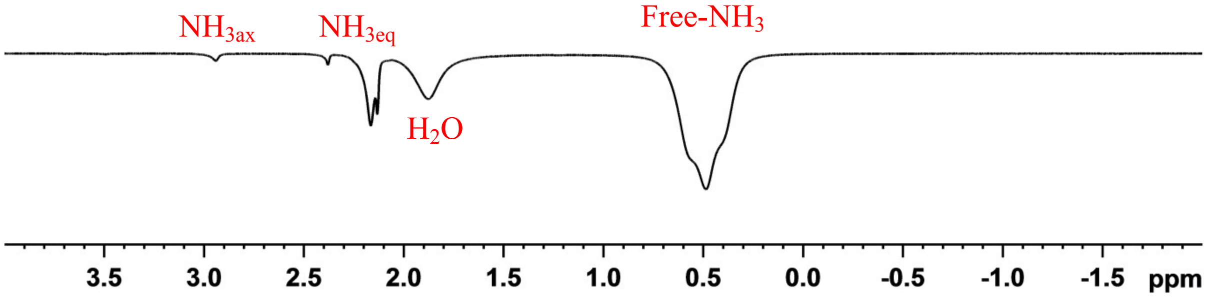

Fig. S2b ^{1}H SABRE enhanced NMR spectrum of ammonia in CD_2Cl_2 (top) and expansion (bottom).

- **Benzylamine**

Fig. S3 reflects a series of single scan ^{1}H NMR spectra of benzylamine: a) ^{1}H NMR spectrum thermally polarised x8 vertical scale, b) ^{1}H SABRE NMR spectrum, c-f) ^{1}H-^{13}C refocussed INEPT ^{13}C SABRE NMR spectra and d) ^{1}H NMR spectrum recorded after adding 1 µl of D_2O (max enhancement (per proton) of H_2O 215 fold); conditions: [IrCl(COD)(IMes)] (5 mM), benzylamine (10 eq.), CD_2Cl_2 (0.6 mL); Substrate concentration: 50 mM. ^{1}H NMR signal enhancement (fold/per proton): 72-(NH_2), 56-(CH_2) and 194-(Ph).

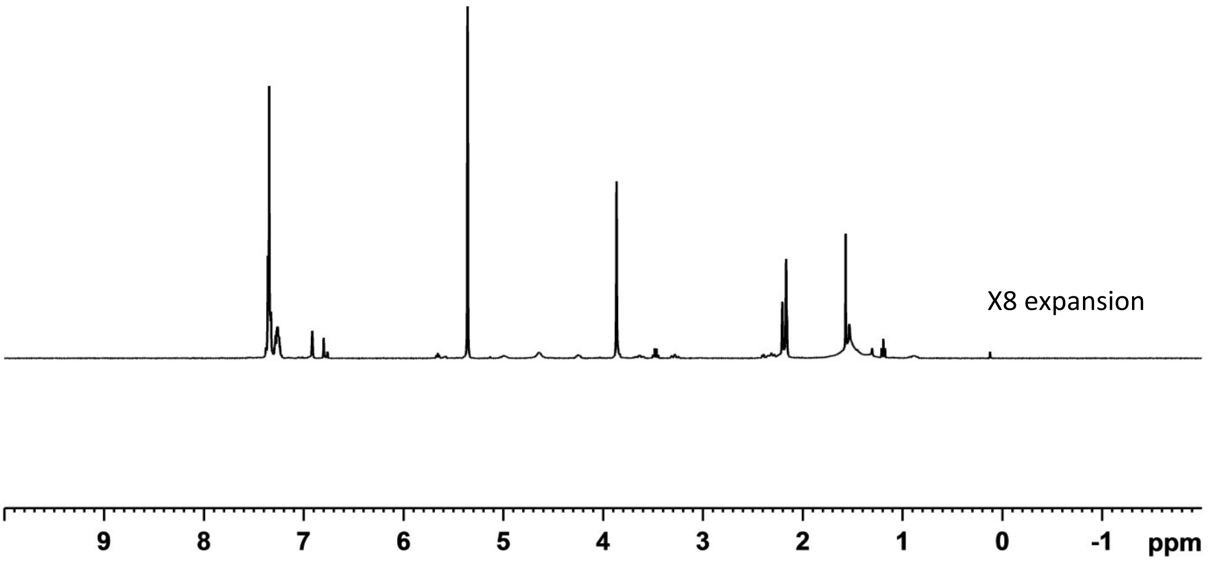

Fig. S3a Thermally polarised ^{1}H NMR reference spectrum of benzylamine

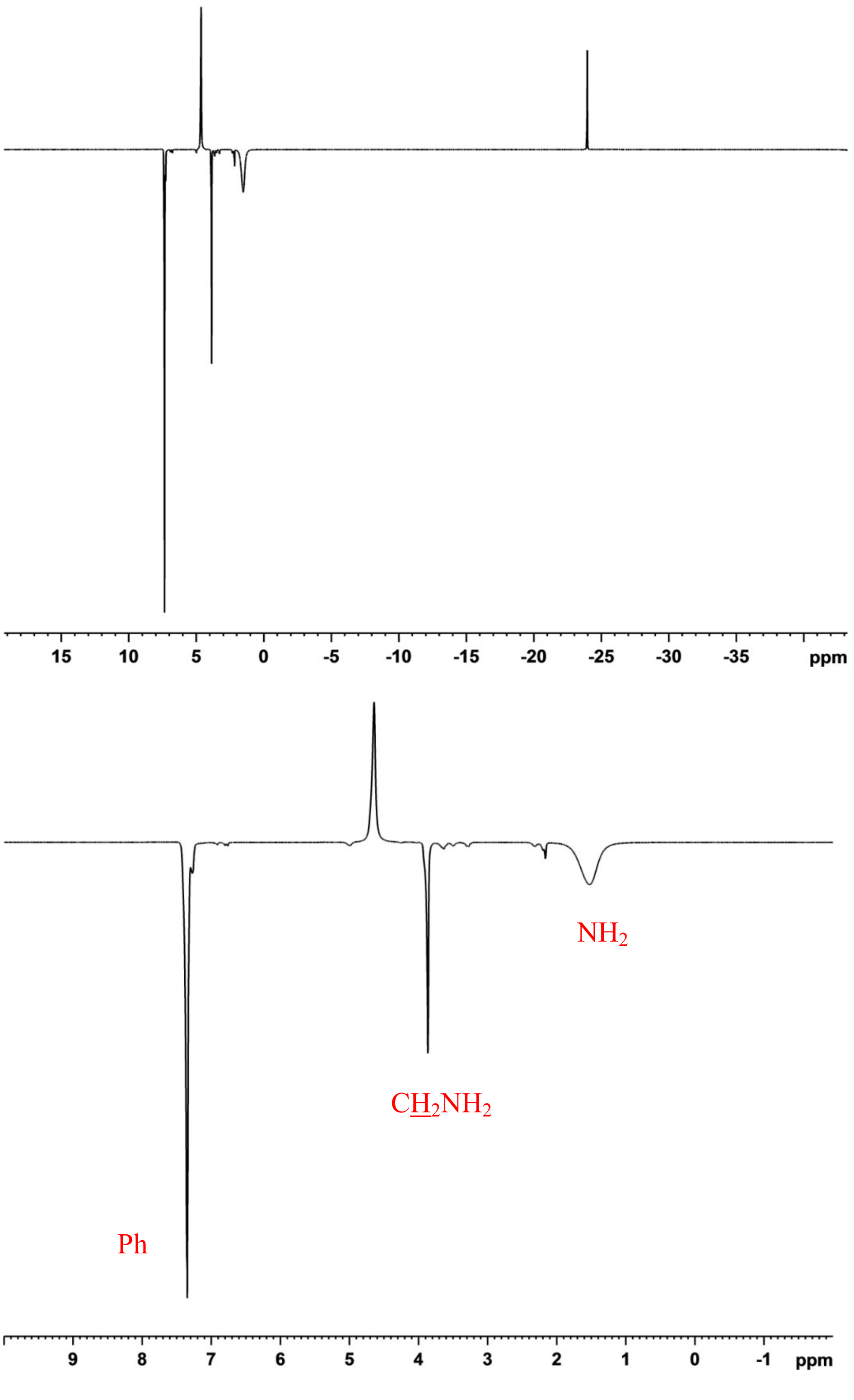

Fig. S3b ^{1}H SABRE enhanced NMR spectrum of benzylamine (top) and expansion (bottom)

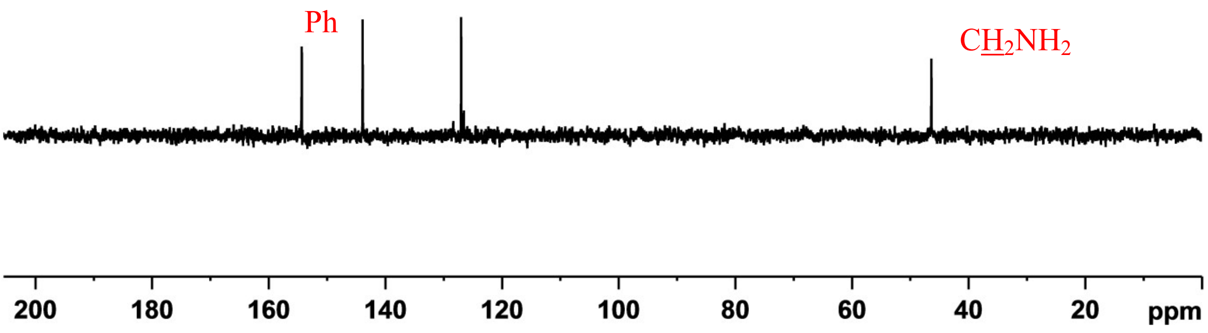

Fig. S3c ^1H-^{13}C refocussed INEPT ^{13}C SABRE enhanced NMR spectrum of benzylamine (J_{XH} = 10 Hz, B = 60 G)

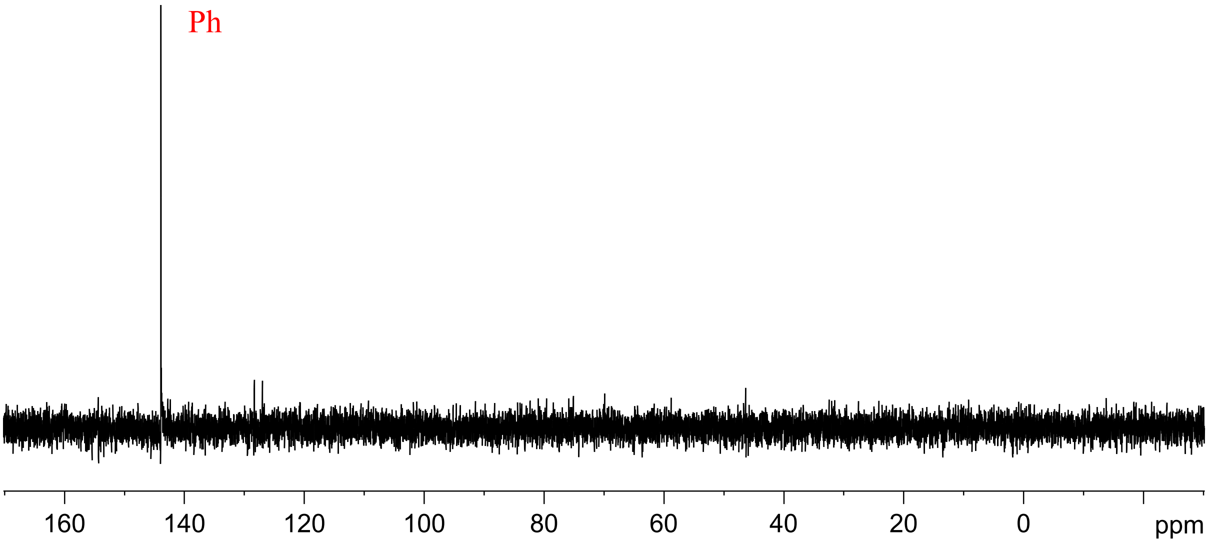

Fig. S3d ^1H-^{13}C refocussed INEPT ^{13}C SABRE enhanced NMR spectrum of benzylamine (J_{XH} = 10 Hz, B = μ-magnetic shield)

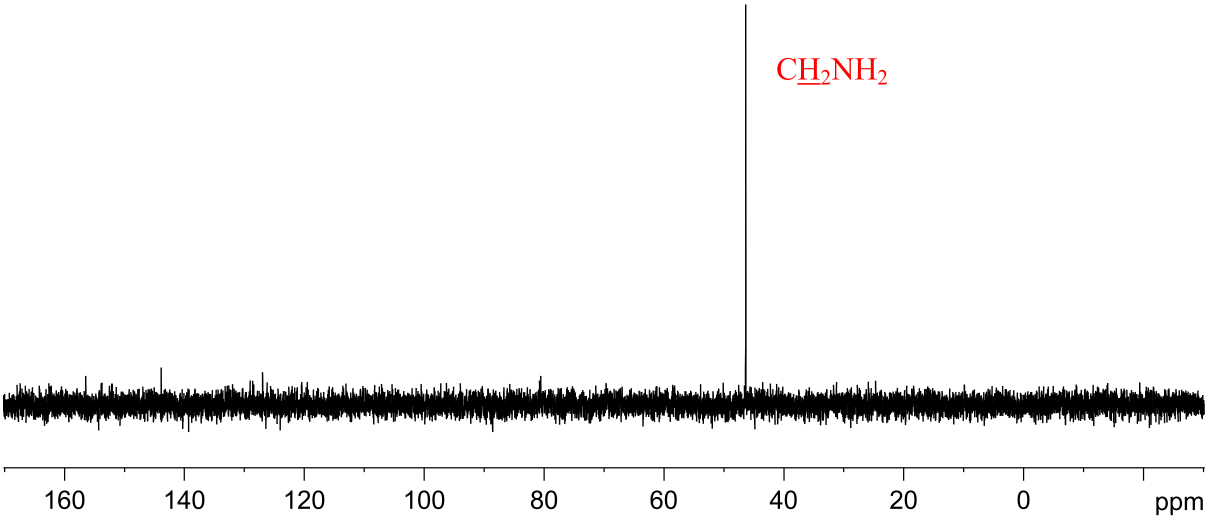

Fig. S3e ^1H-^{13}C refocussed INEPT ^{13}C SABRE enhanced NMR spectrum of benzylamine (J_{XH} = 135 Hz, B = 60 G)

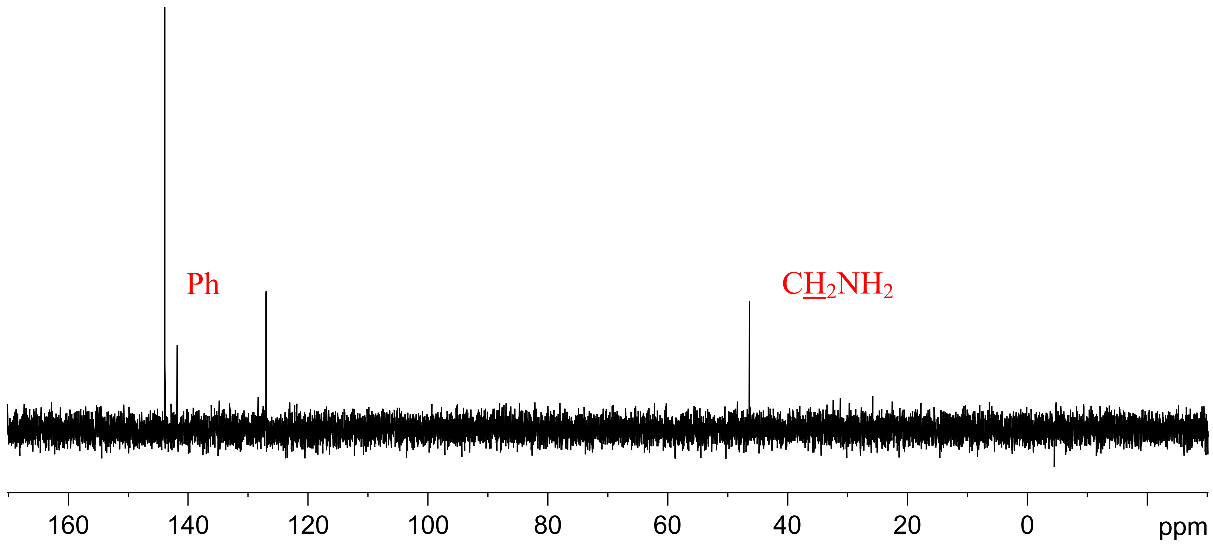

Fig. S3f ^{1}H-^{13}C refocussed INEPT ^{13}C SABRE enhanced NMR spectrum of benzylamine (J_{XH} = 135 Hz, B = μ-magnetic shield)

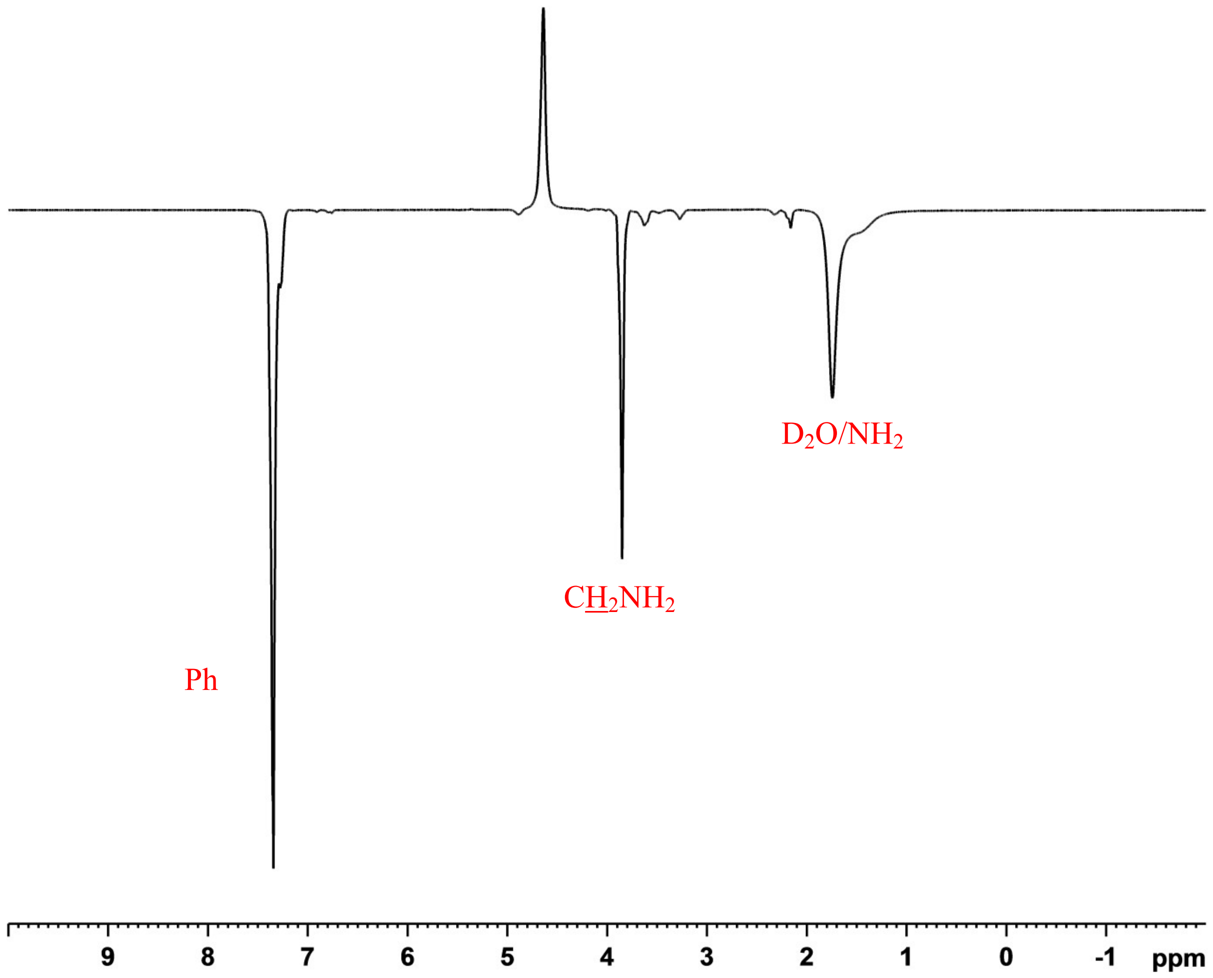

Fig. S3g ^{1}H SABRE enhanced NMR spectrum for benzylamine after adding 1 μl of D_2O

- **Benzylamine-^{15}N**

Fig. S4 reflects a series of single scan NMR spectra of benzylamine-^{15}N: a) ^{1}H NMR spectrum thermally polarised x16, b) ^{1}H SABRE NMR spectrum and c) ^{15}N SABRE NMR spectrum using conditions: [IrCl(COD)(IMes)] (5 mM), benzylamine-^{15}N (10 eq.), CD_2Cl_2 (0.6 mL); Substrate concentration: 50 mM. ^{1}H enhancement (fold/per proton): 33-(NH_2), 34-(CH_2) and 52-(Ph)

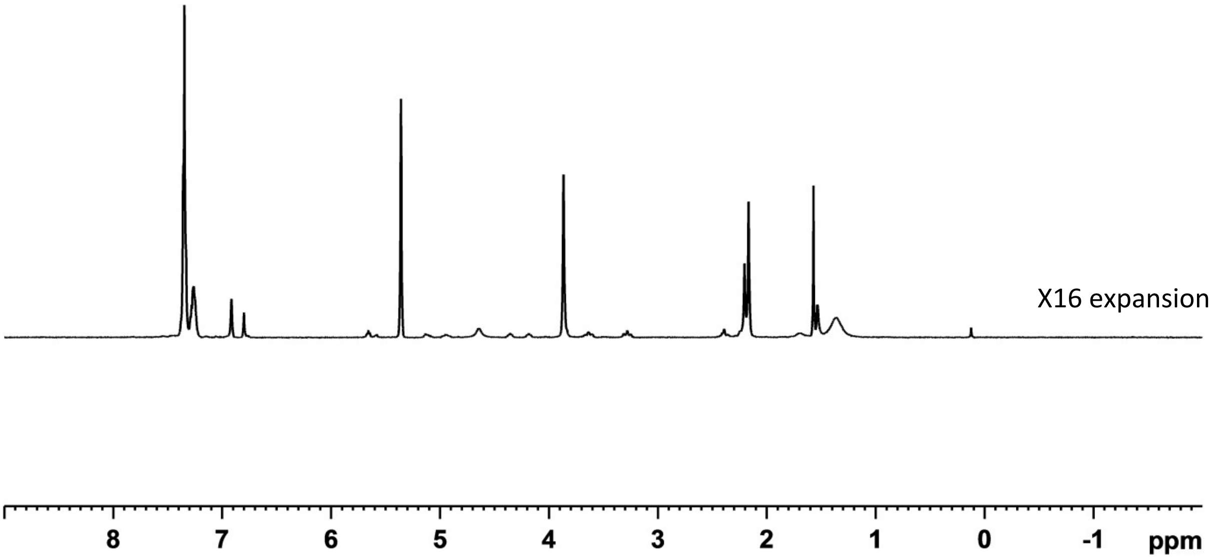

Fig. S4a Thermally polarised ^{1}H NMR reference spectrum of benzylamine-^{15}N

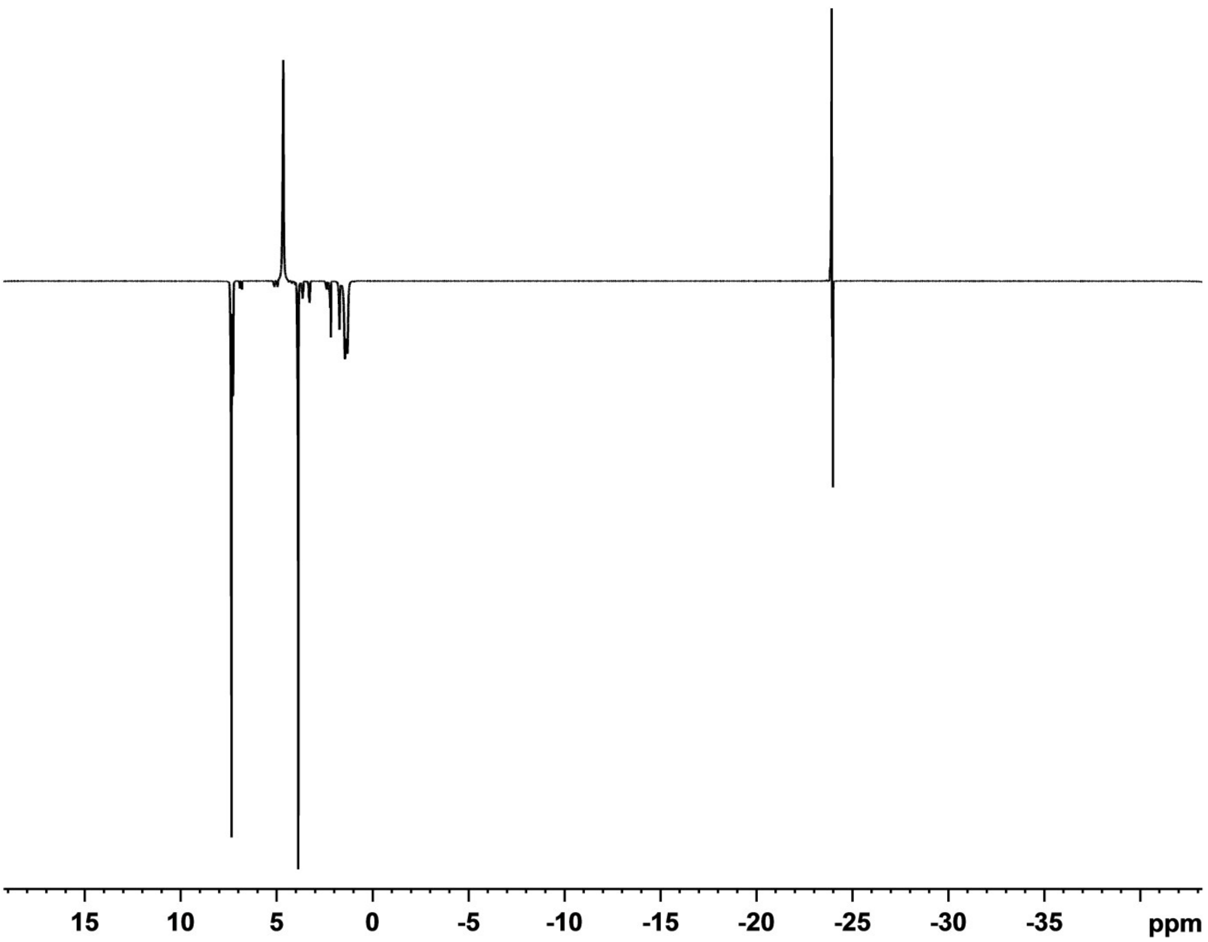
15
10
5
0
-5
-10
-15
-20
-25
-30
-35
ppm

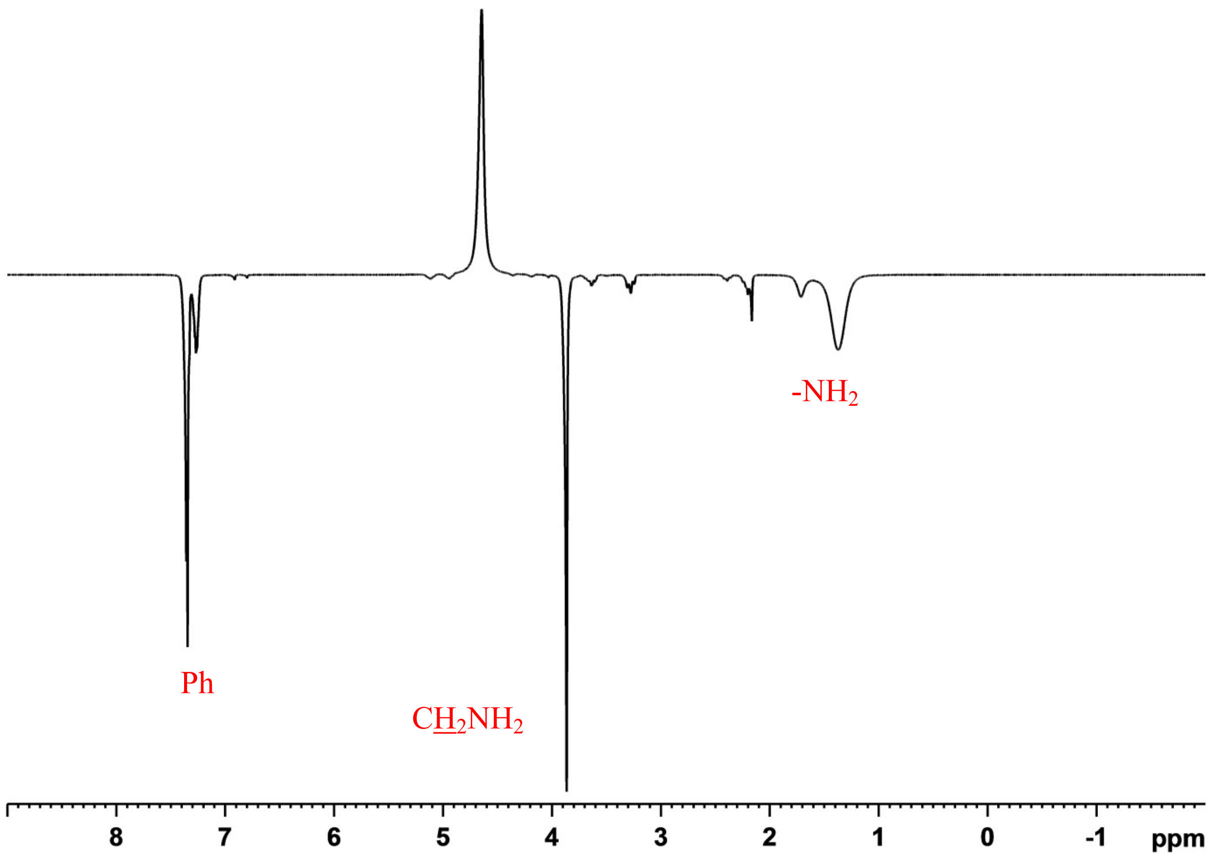
-NH2
Ph
CH2NH2
8
7
6
5
4
3
2
1
0
-1
ppm

Fig. S4b ^{1}H SABRE enhanced NMR spectrum of benzylamine-^{15}N (top) and expansion (bottom)

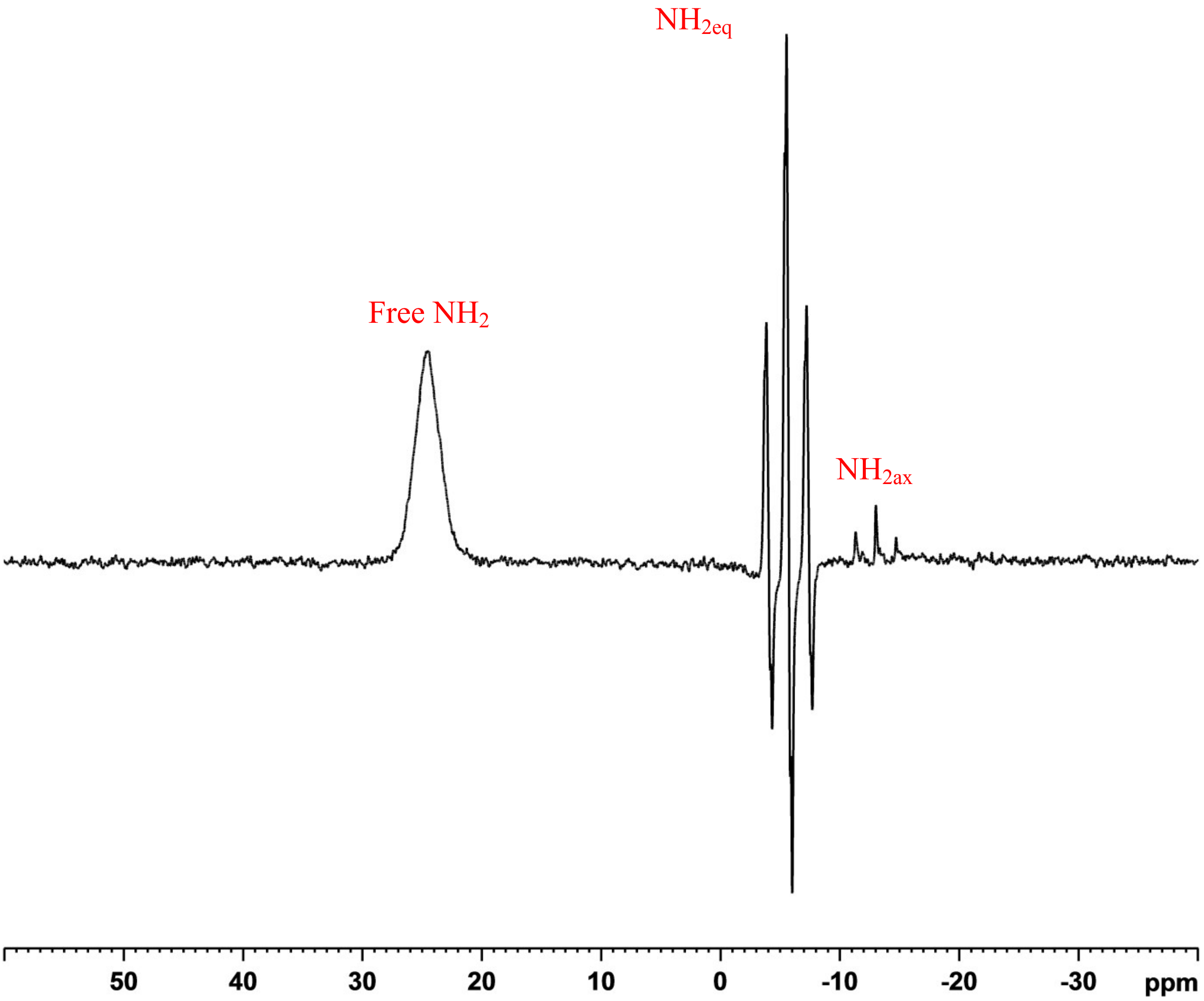

Fig. S4c ^{15}N SABRE enhanced NMR spectrum of benzylamine-^{15}N (B = 0 G)

- **Phenylethylamine**

Fig. S5 reflects a series of single scan NMR spectra of phenylethylamine: a) ^{1}H NMR spectrum thermally polarised x8, b) ^{1}H SABRE NMR spectrum and c) INEPT ^{13}C SABRE NMR spectrum using conditions: [IrCl(COD)(IMes)] (5 mM), phenylethylamine (10 eq.), CD_2Cl_2 (0.6 mL); Substrate concentration: 50 mM. ^{1}H enhancement (fold/per proton): 108-(NH_2), 45- (CH_2), 50-(NCH_2) and 92-(Ph)

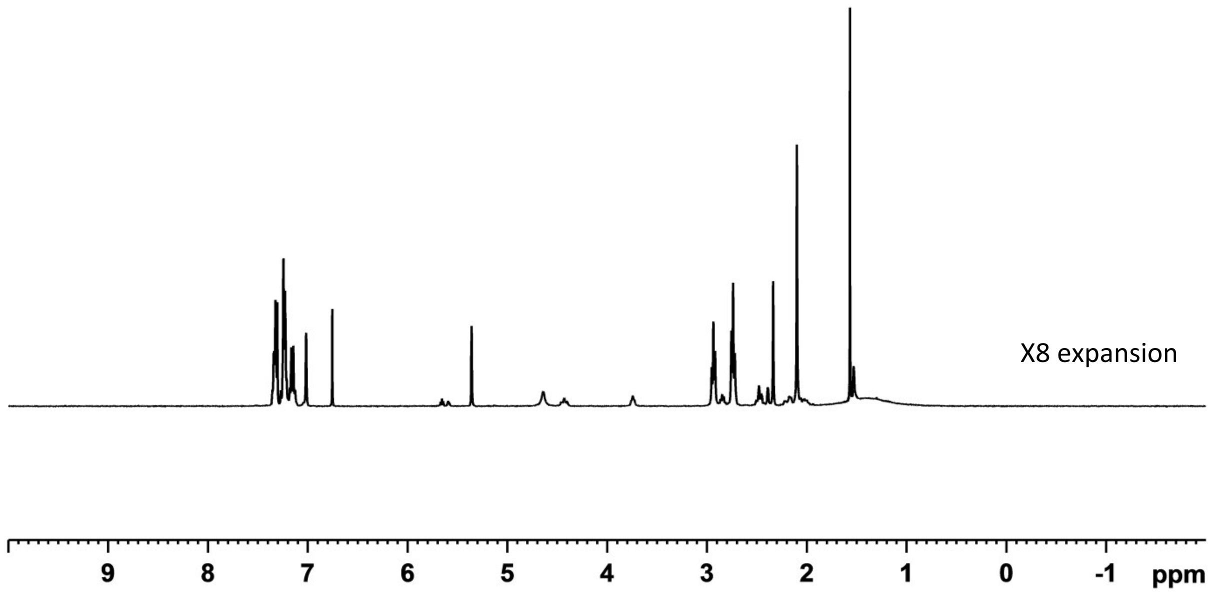

Fig. S5a Thermally polarised ^{1}H NMR reference spectrum for phenylethylamine

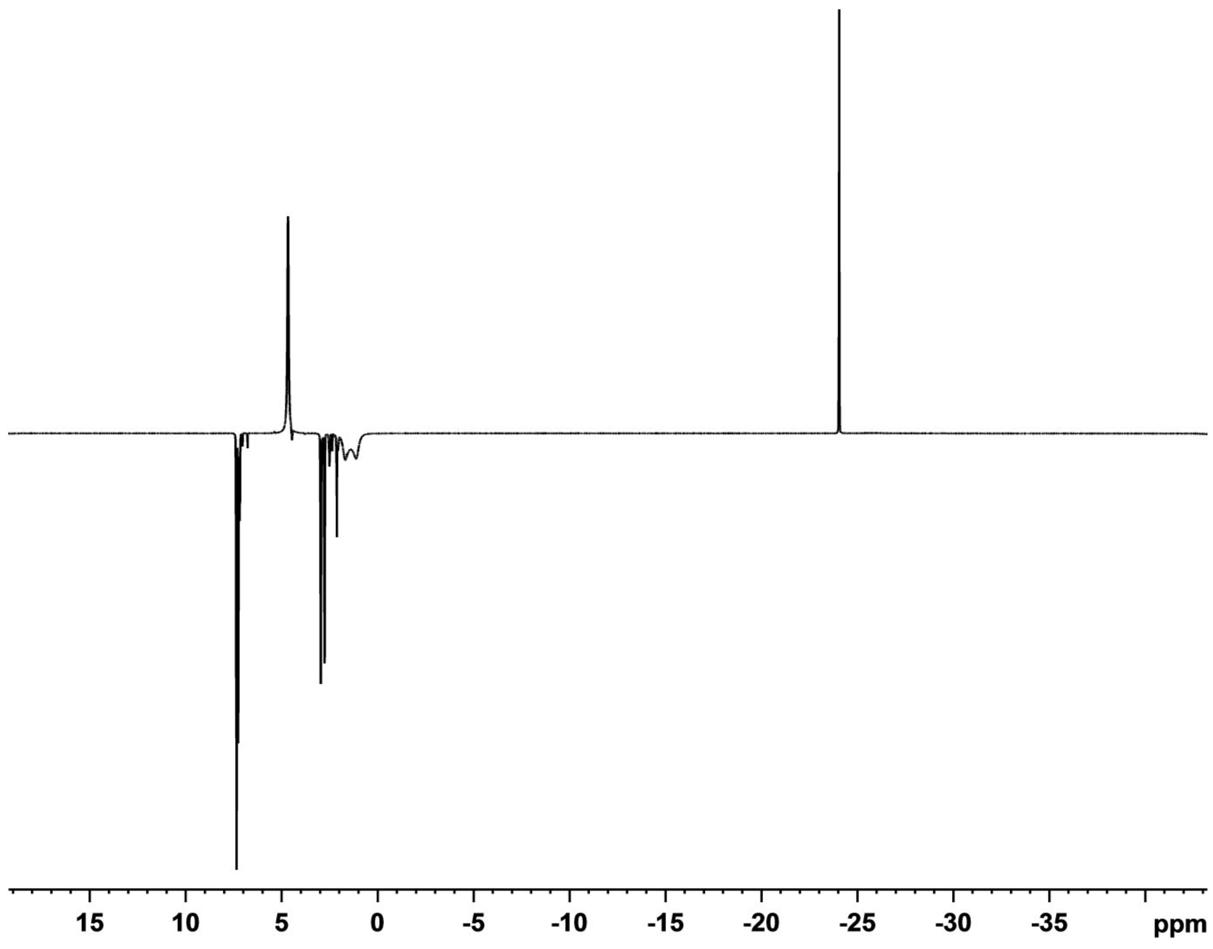

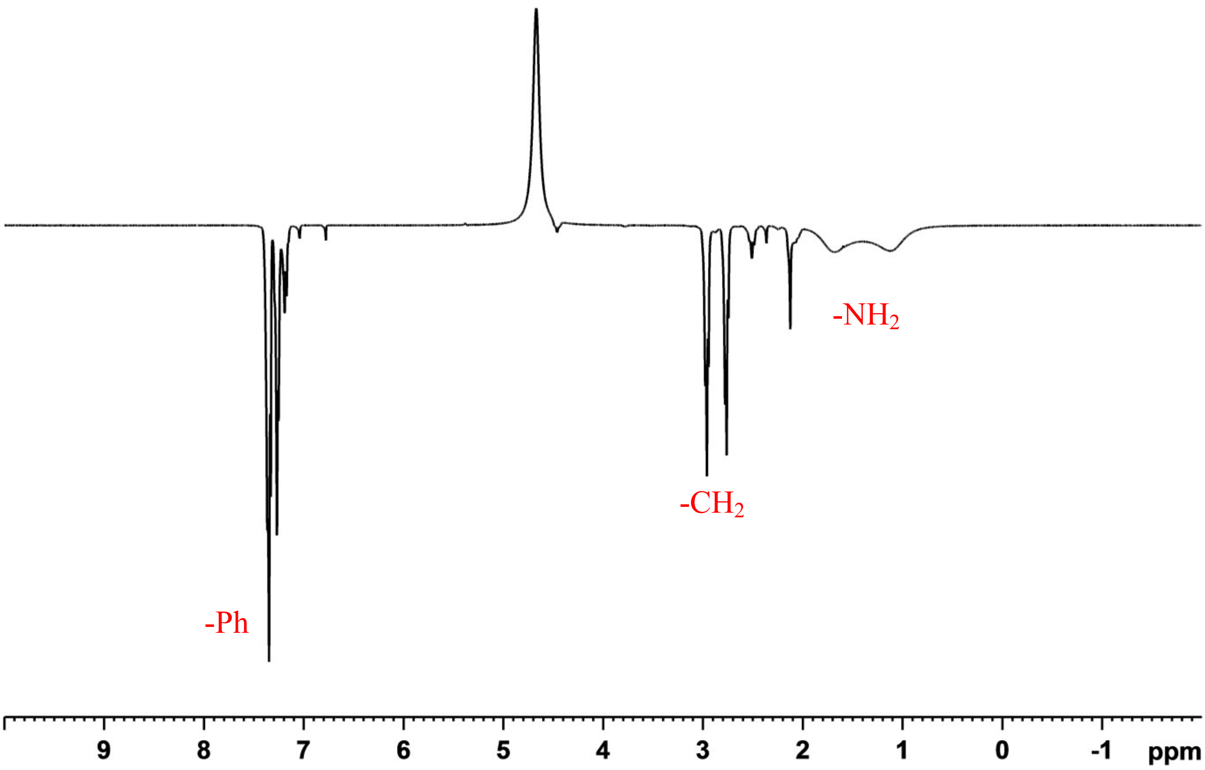

Fig. S5b ^{1}H SABRE enhanced NMR spectrum of phenylethylamine (top) and expansion (bottom)

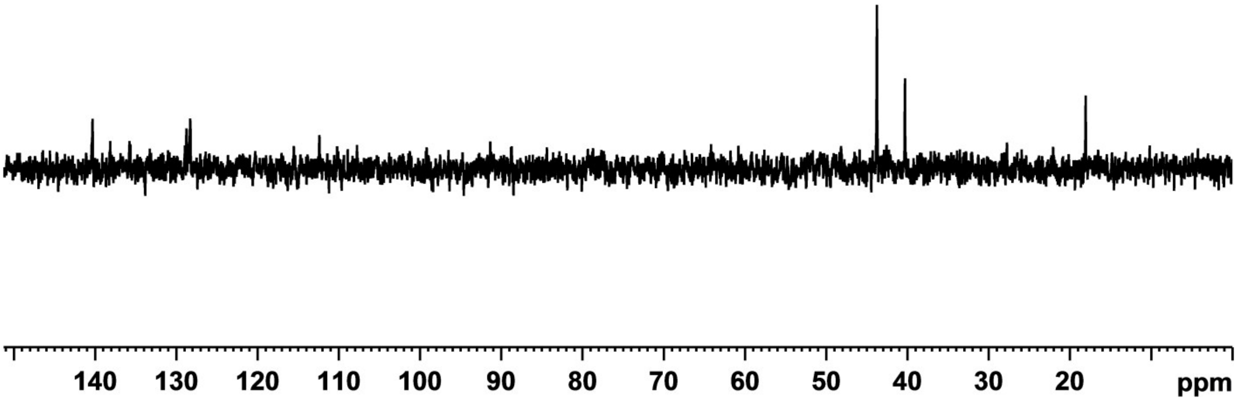

Fig. S5c INEPT ^{13}C SABRE enhanced NMR spectrum of phenylethylamine (J_{XH} = 122 Hz, B = 60 G)

- **Phenoxyethylamine**

Fig. S6 reflects a series of single scan NMR spectra of phenoxyethylamine: a) ^{1}H NMR spectrum thermally polarised x16, b) ^{1}H SABRE NMR spectrum and c) INEPT ^{13}C SABRE NMR spectrum using conditions: [IrCl(COD)(IMes)] (5 mM), phenoxyethylamine (10 eq.), CD_2Cl_2 (0.6 mL); Substrate concentration: 50 mM. ^{1}H enhancement (fold/per proton): 99-(N$\underline{H}_2$), 47- (NC$\underline{H}_2$), 147-(C$\underline{H}_2$O) and 8-(Ph)

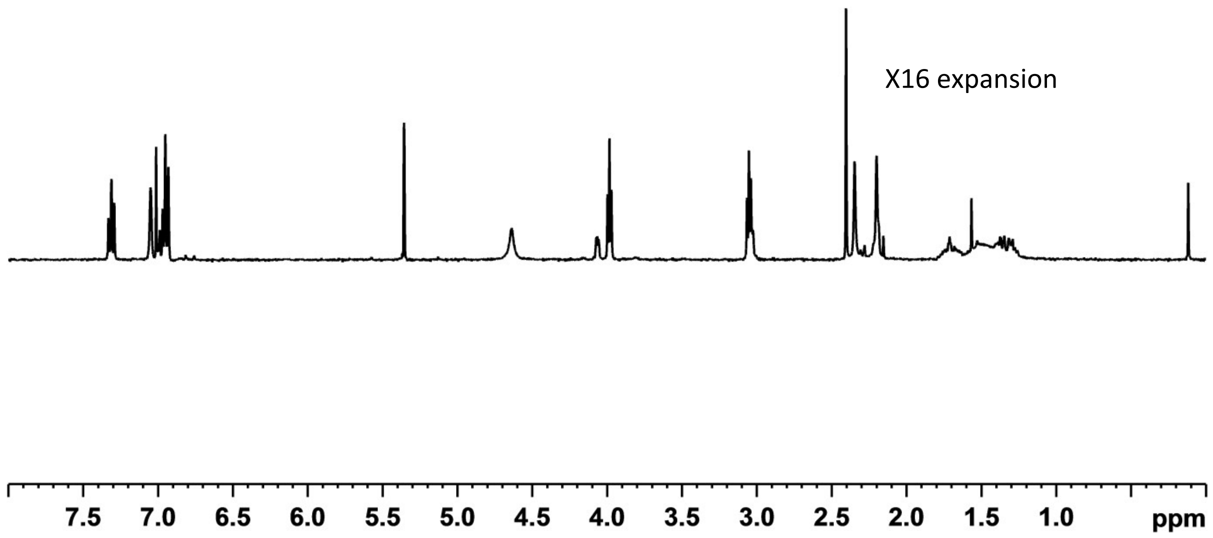

Fig. S6a Thermally polarised ^{1}H NMR reference spectrum of phenoxyethylamine

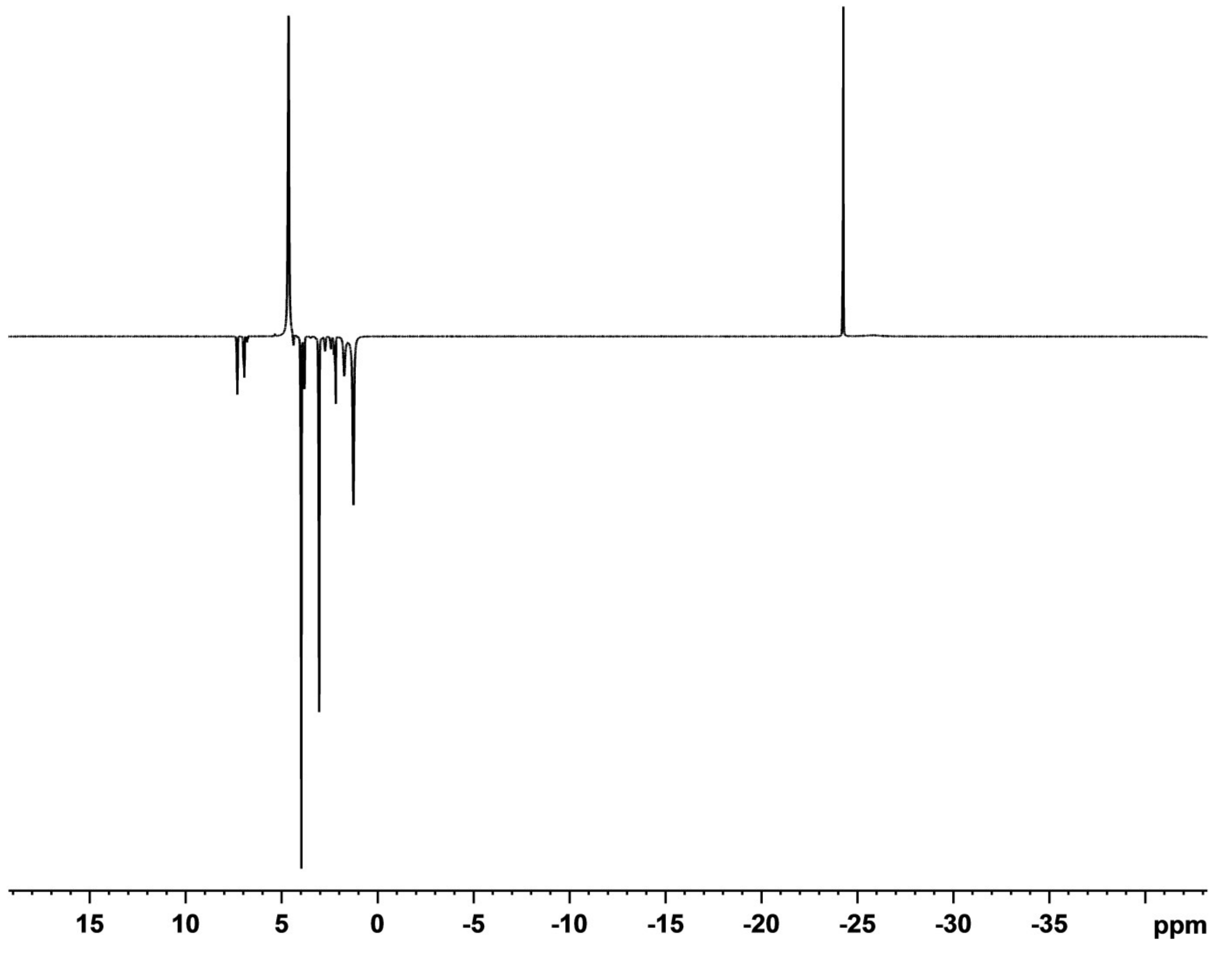

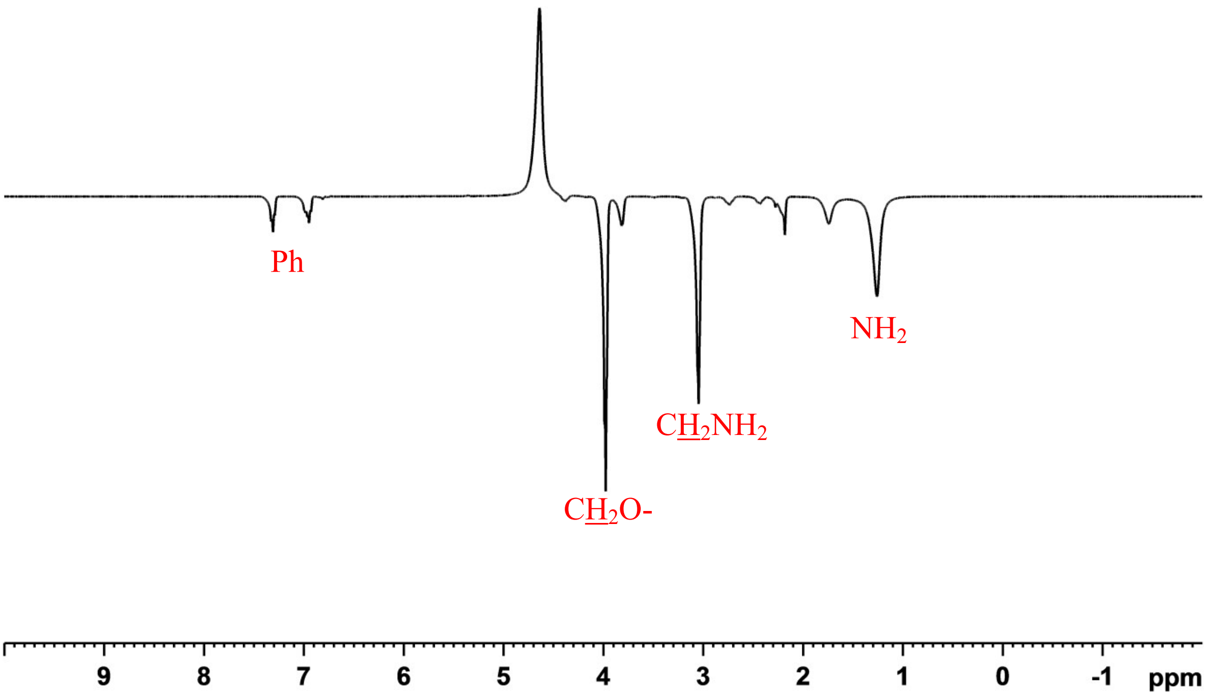

Fig. S6b ^{1}H SABRE enhanced NMR spectrum of phenoxyethylamine (top) and expansion (bottom)

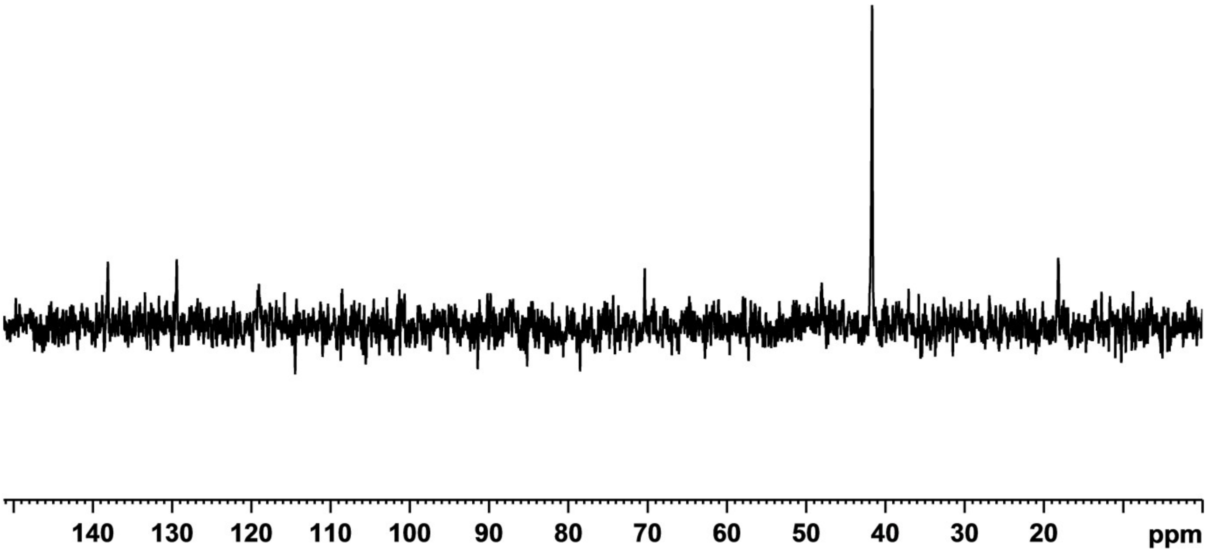

Fig. S6c INEPT ^{13}C SABRE enhanced NMR spectrum of phenoxyethylamine (J_{XH} = 165 Hz, B = 60 G)

- **Isobutylamine**

Fig. S7 reflects a series of single scan NMR spectra of isobutylamine: a) ^{1}H NMR spectrum thermally polarised x8 and b) ^{1}H SABRE NMR spectrum using conditions: [IrCl(COD)(IMes)] (5 mM), isobutylamine (10 eq.), CD_2Cl_2 (0.6 mL); Substrate

concentration: 50 mM. ^{1}H enhancement (fold/per proton): 187- (N<u>H</u>$_2$), 83-(-C<u>H</u>$_2$), 65-(C<u>H-</u>) and 124-(-CH$_3$)

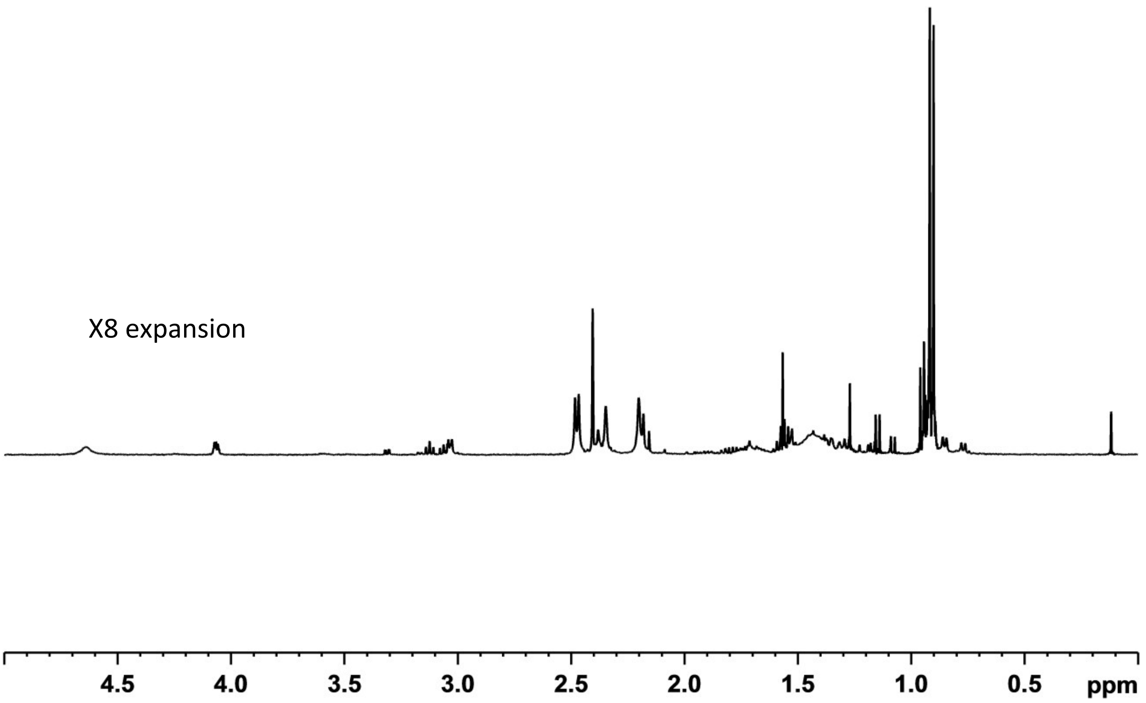

Fig. S7a Thermally polarised ^{1}H NMR reference spectrum for isobutylamine

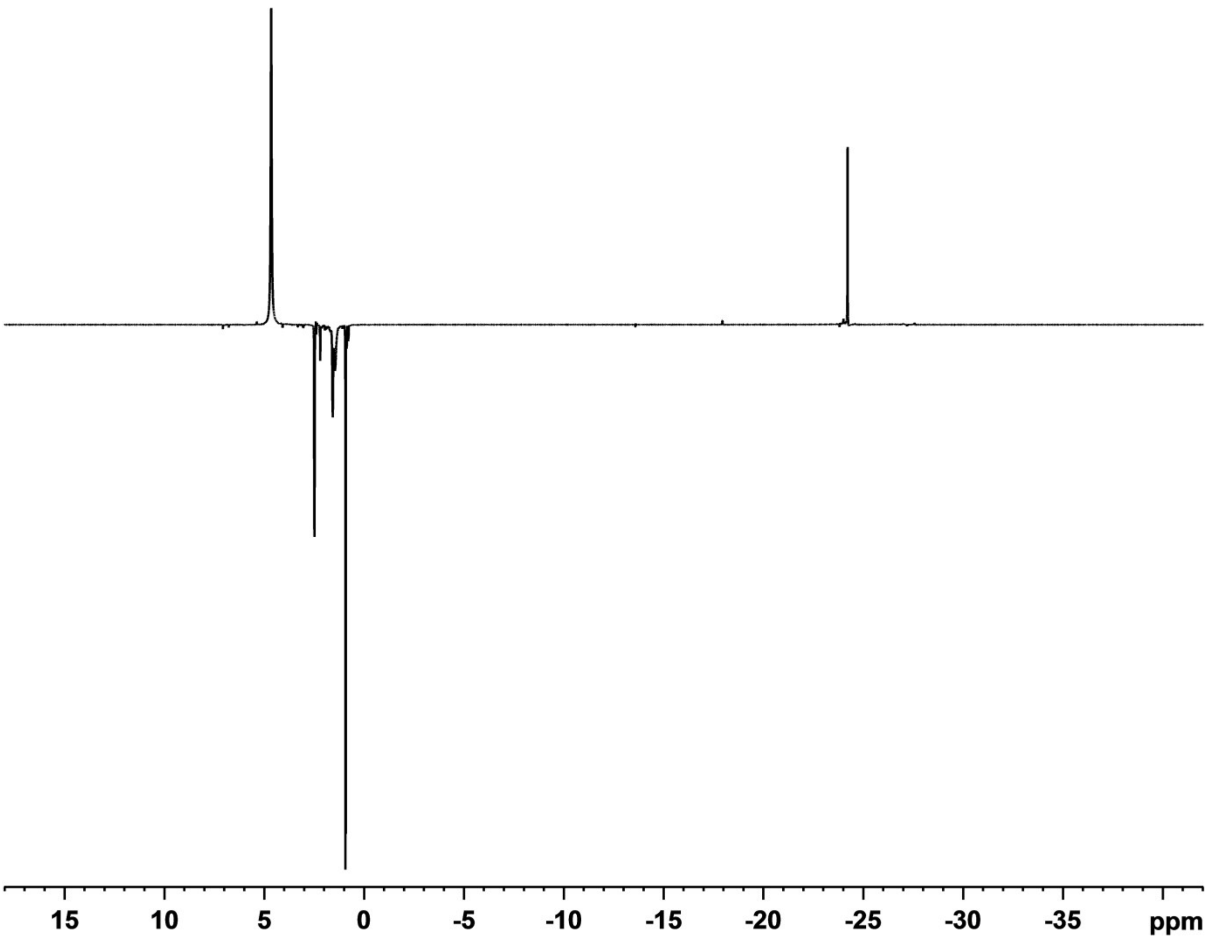

15 10 5 0 -5 -10 -15 -20 -25 -30 -35
ppm

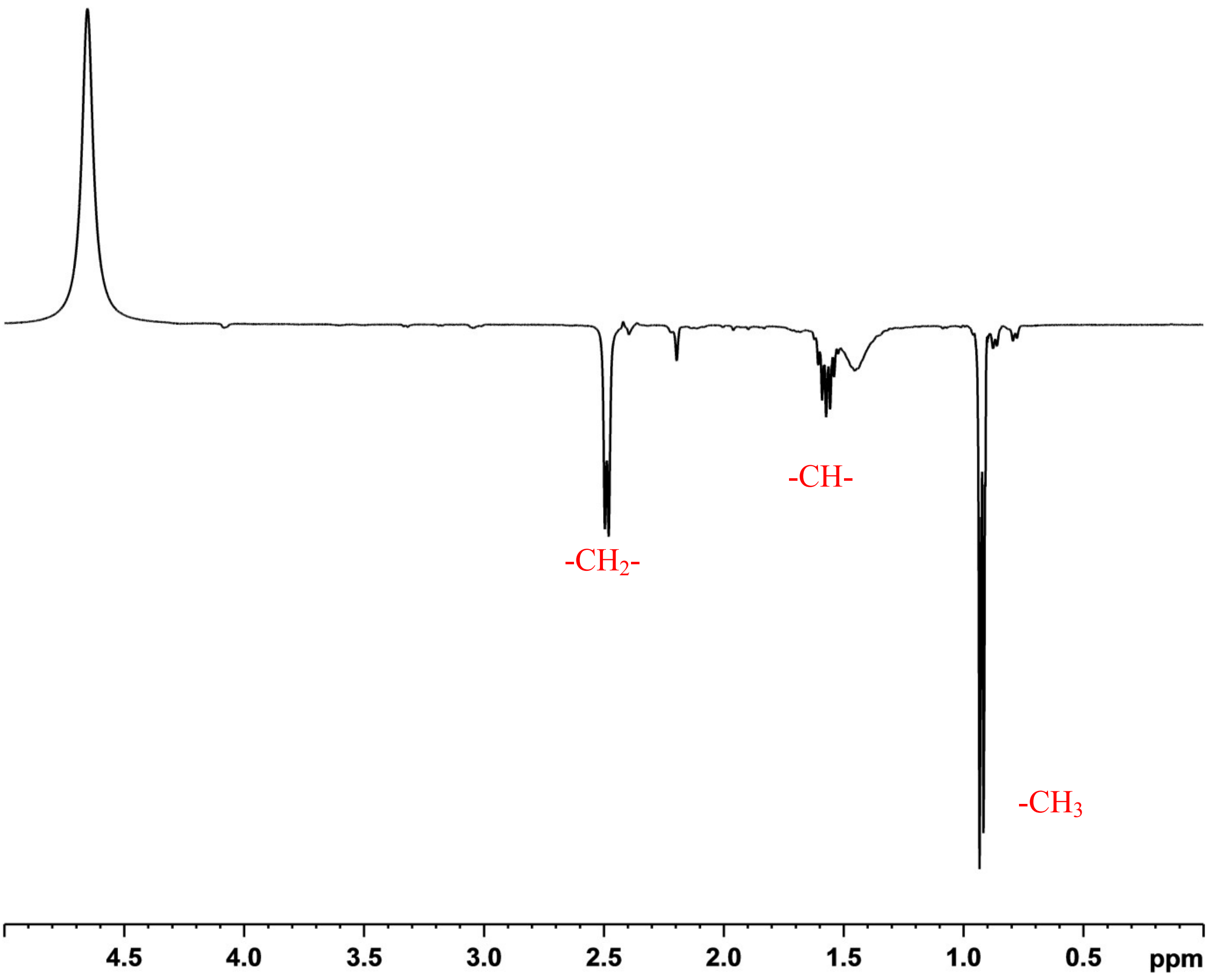

Fig. S7b ^{1}H SABRE enhanced NMR spectrum of isobutylamine (top) and expansion (bottom)

- **Allylamine**

Fig. S8 reflects a series of single scan NMR spectra of allylamine: a) ^{1}H NMR spectrum thermally polarised x4 and b) ^{1}H SABRE NMR spectrum using conditions: [IrCl(COD)(IMes)] (5 mM), allylamine (10 eq.), CD_2Cl_2 (0.6 mL); Substrate concentration: 50 mM. ^{1}H enhancement (fold/per proton): 76-(N$\underline{H}_2$), 131-(-C$\underline{H}_2$N), 178-(C$\underline{H}$-) and 158-(-CH_2)

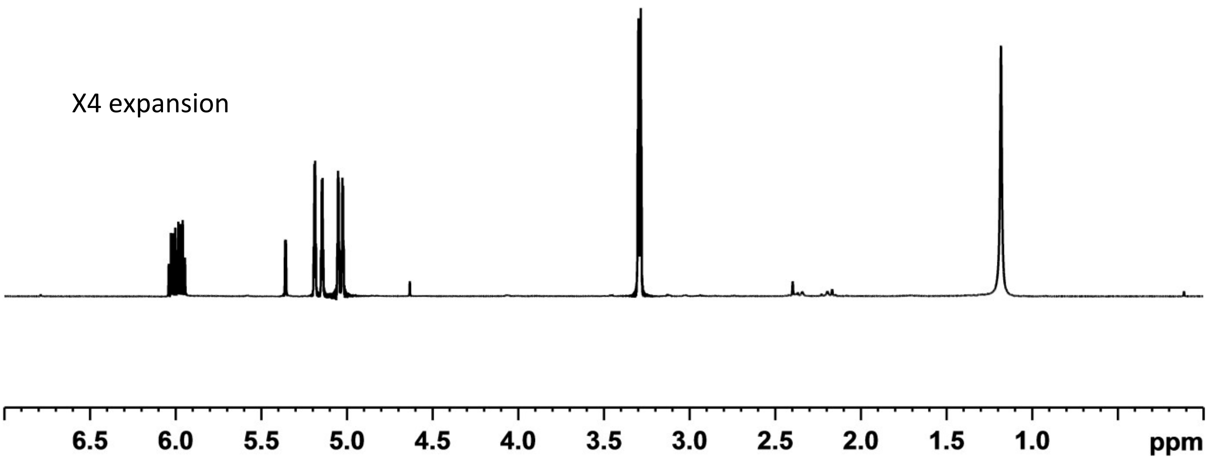

Fig. S8a Thermally polarised ^{1}H NMR reference spectrum of allylamine

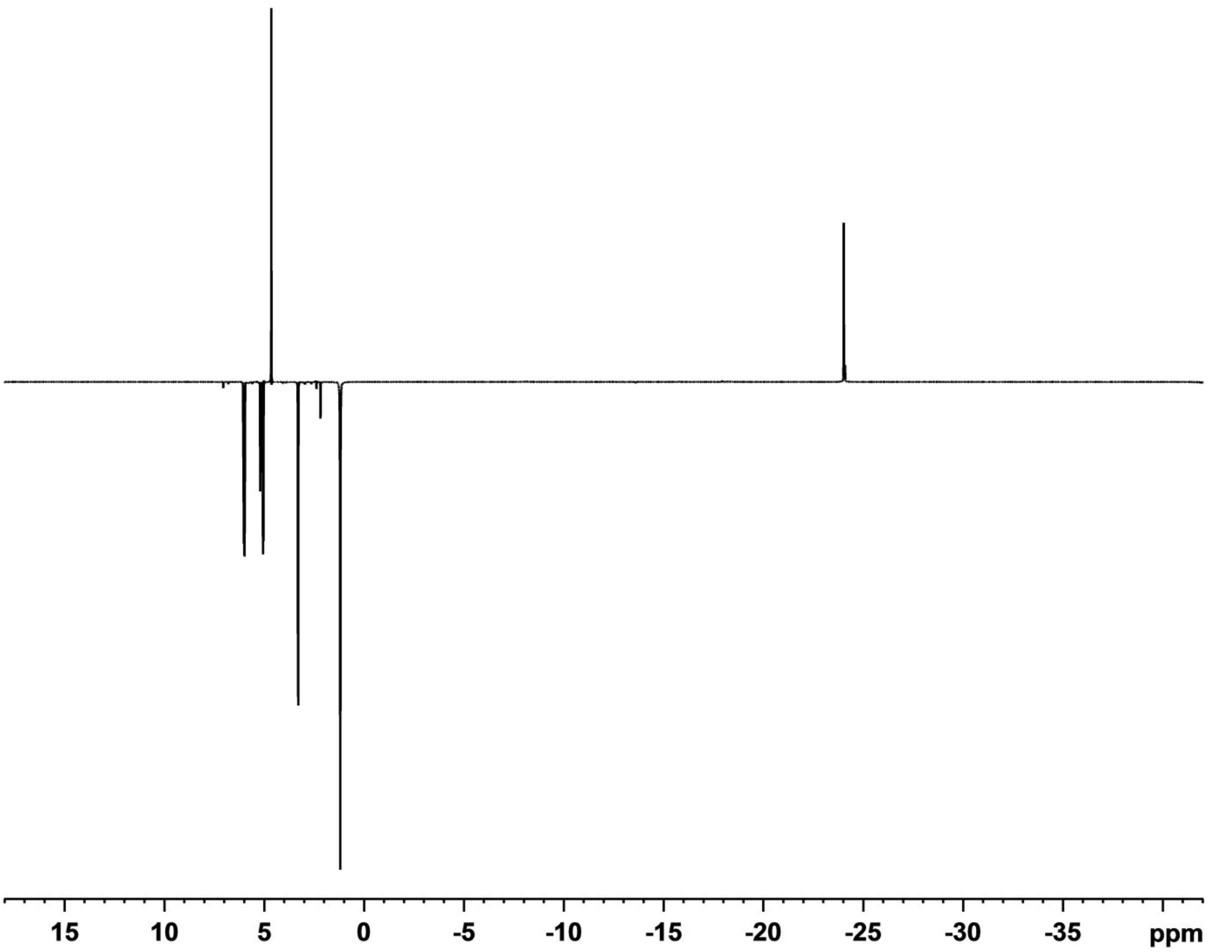

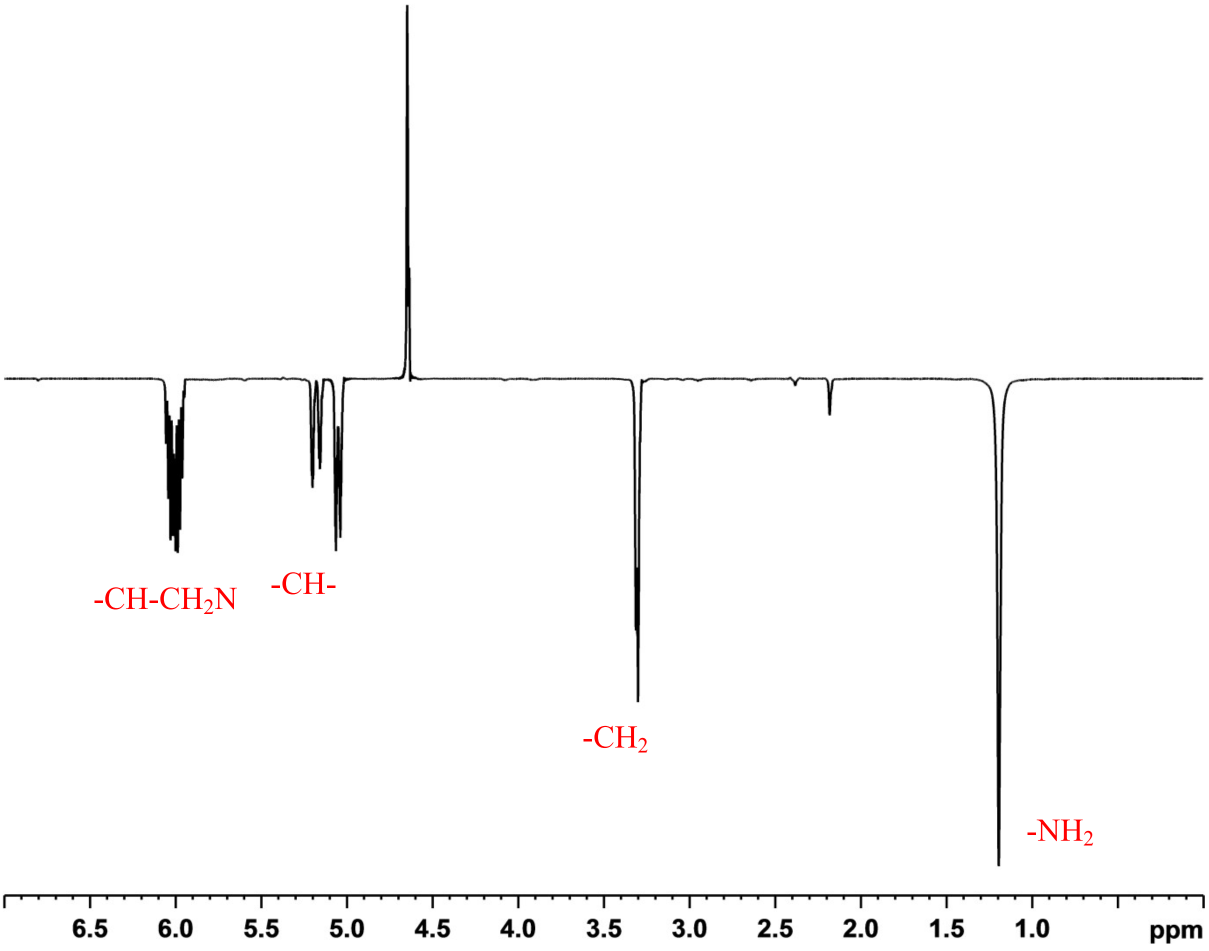

Fig. S8b ^{1}H SABRE enhanced NMR spectrum of allylamine (top) and expansion (bottom)

- **Tryptamine**

Fig. S9 reflects a series of single scan NMR spectra of tryptamine: a) ^{1}H NMR spectrum thermally polarised x8 and b) ^{1}H SABRE NMR spectrum using conditions: [IrCl(COD)(IMes)] (5 mM), tryptamine (10 eq.), CD_2Cl_2 (0.6 mL); Substrate concentration: 50 mM. ^{1}H enhancement (fold/per proton): 21-(-NH_2), 21-(CH_2-N), 8-(-CH_2-), 14-(-CH-), 7-(-NH-) and 28-(Ph)

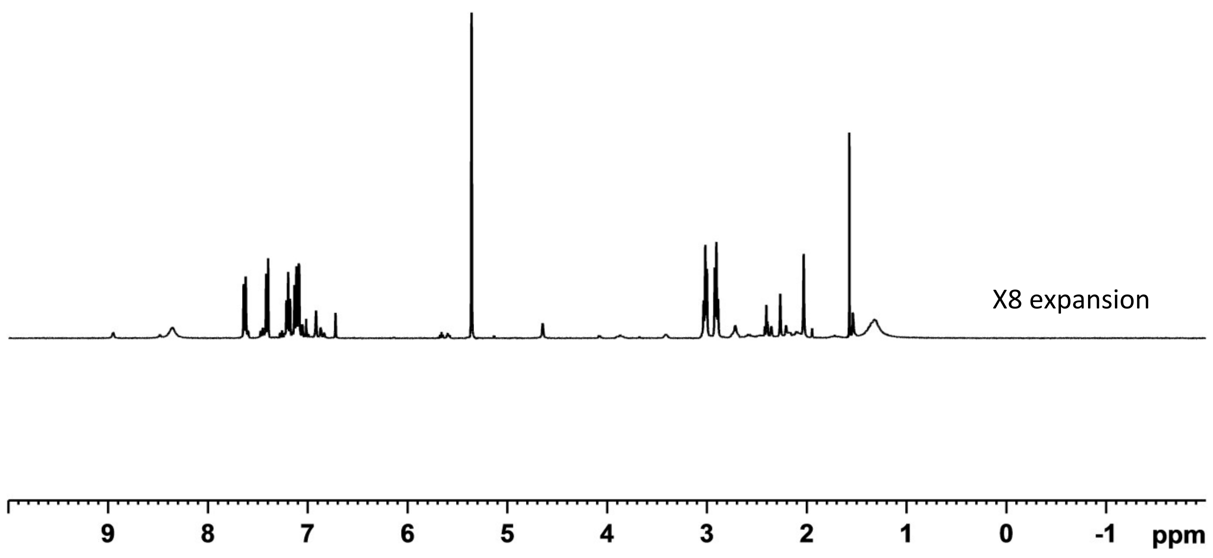

Fig. S9a Thermally polarised ^{1}H NMR reference spectrum of tryptamine

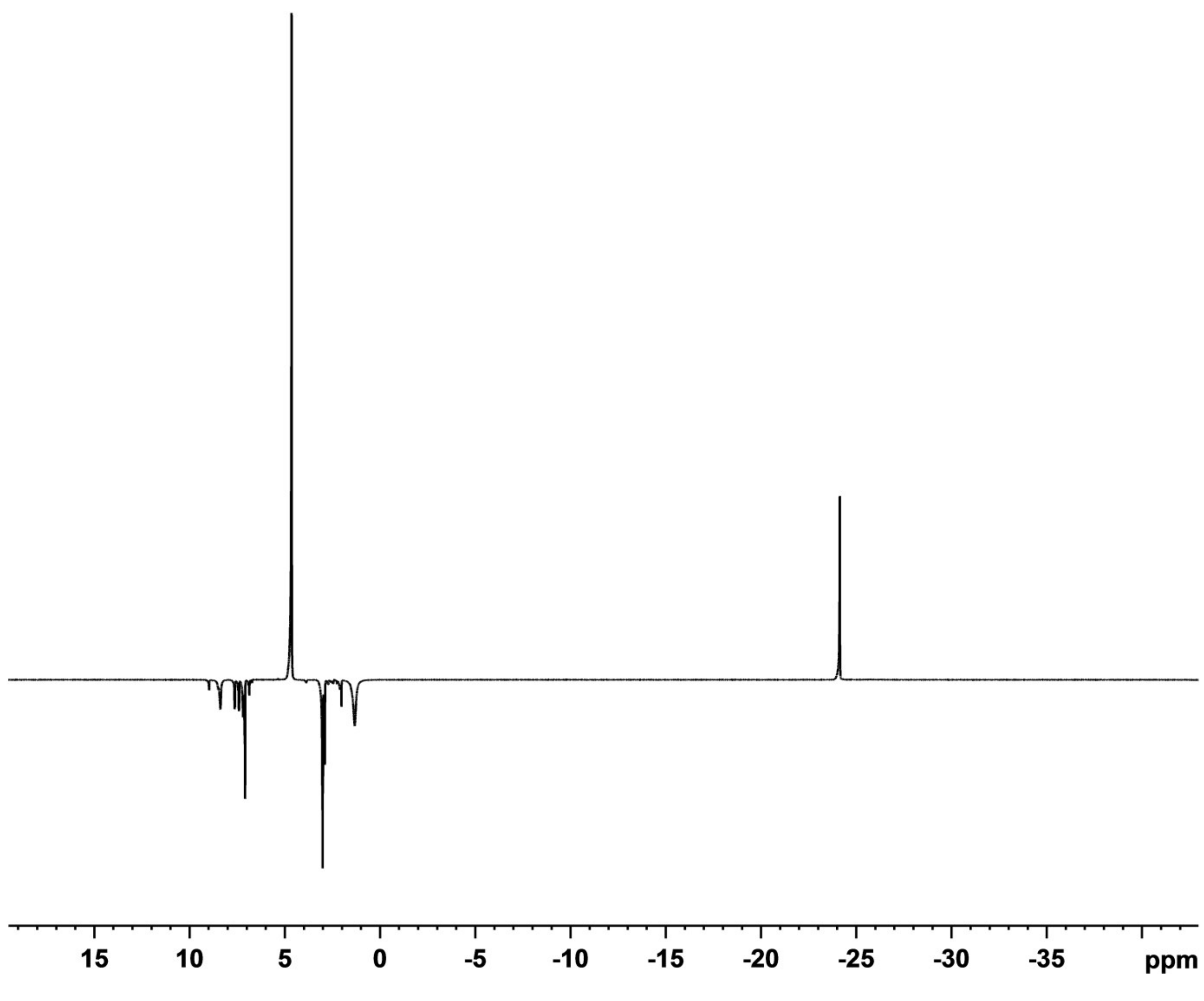

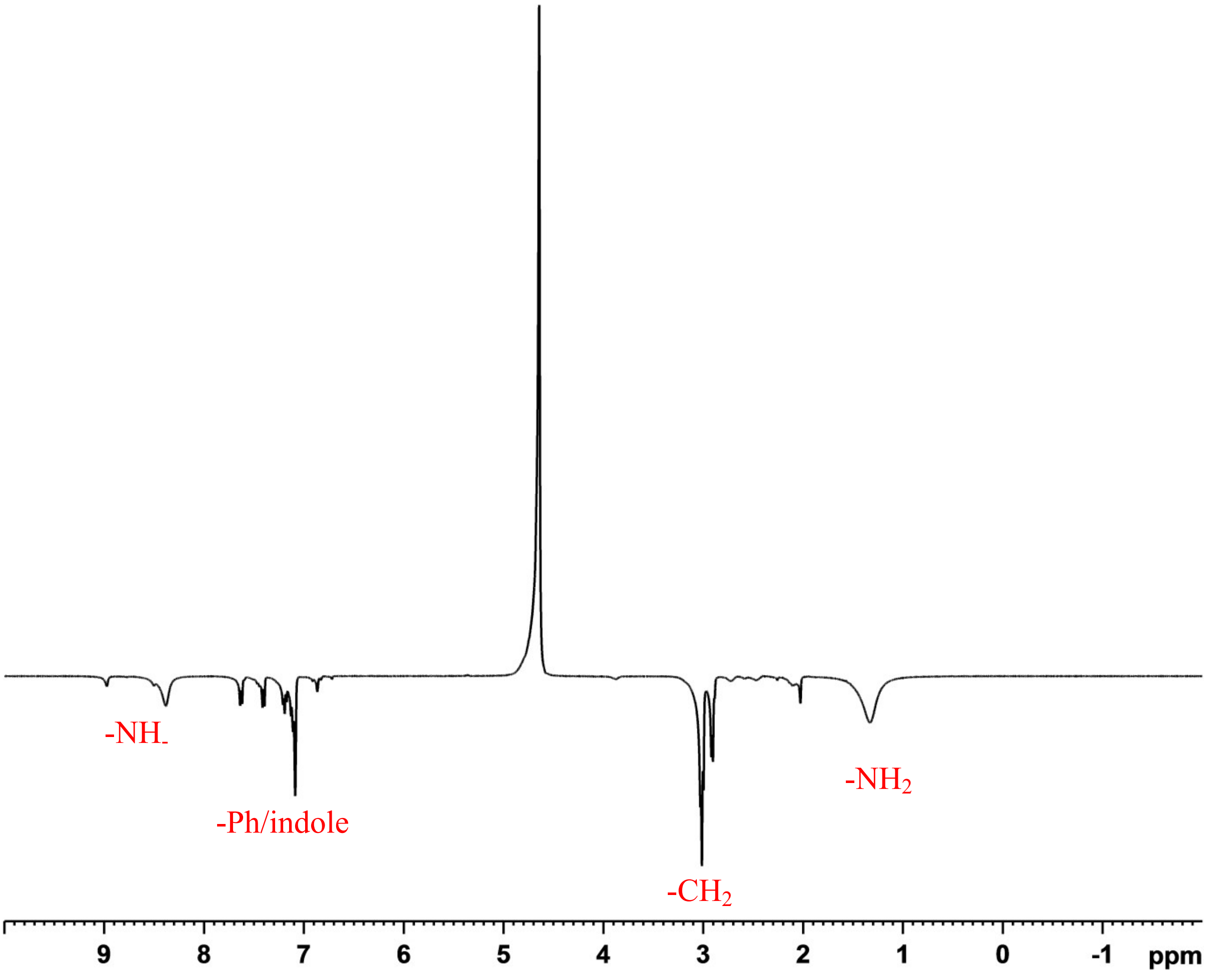

Fig. S9b ^{1}H SABRE enhanced NMR spectrum of tryptamine (top) and expansion (bottom)

3 Representative SABRE NMR spectra and enhancement factors using a co-ligand

- **Aniline**

Fig. S10 reflects a series of single scan NMR spectra of aniline: a) ^{1}H NMR spectrum thermally polarised x8, b) ^{1}H SABRE NMR spectrum c) ^{13}C SABRE NMR spectrum and d) INEPT ^{13}C SABRE NMR spectrum using conditions: [IrCl(COD)(IMes)] (5 mM), aniline (10 eq.), CD_2Cl_2 (0.6 mL); Substrate concentration: 50 mM and MeCN as co-ligand (8 fold excess). ^{1}H enhancement (fold/per proton): 306-(NH_2) and 193-(Ph)

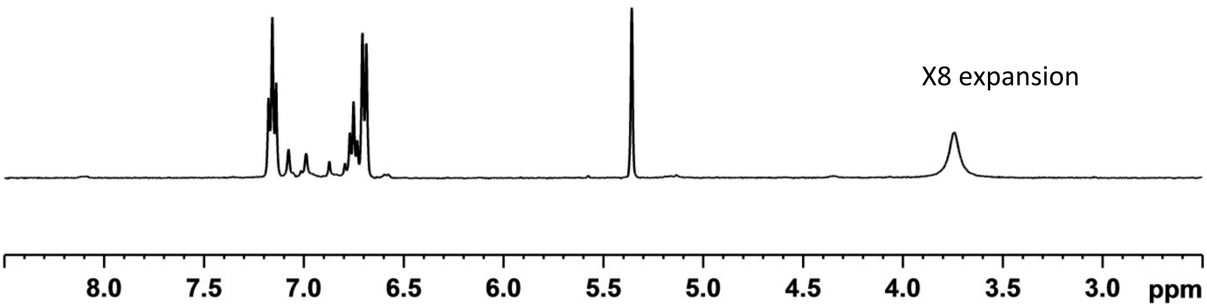

Fig. S10a Thermally polarised ^{1}H NMR reference spectrum of aniline

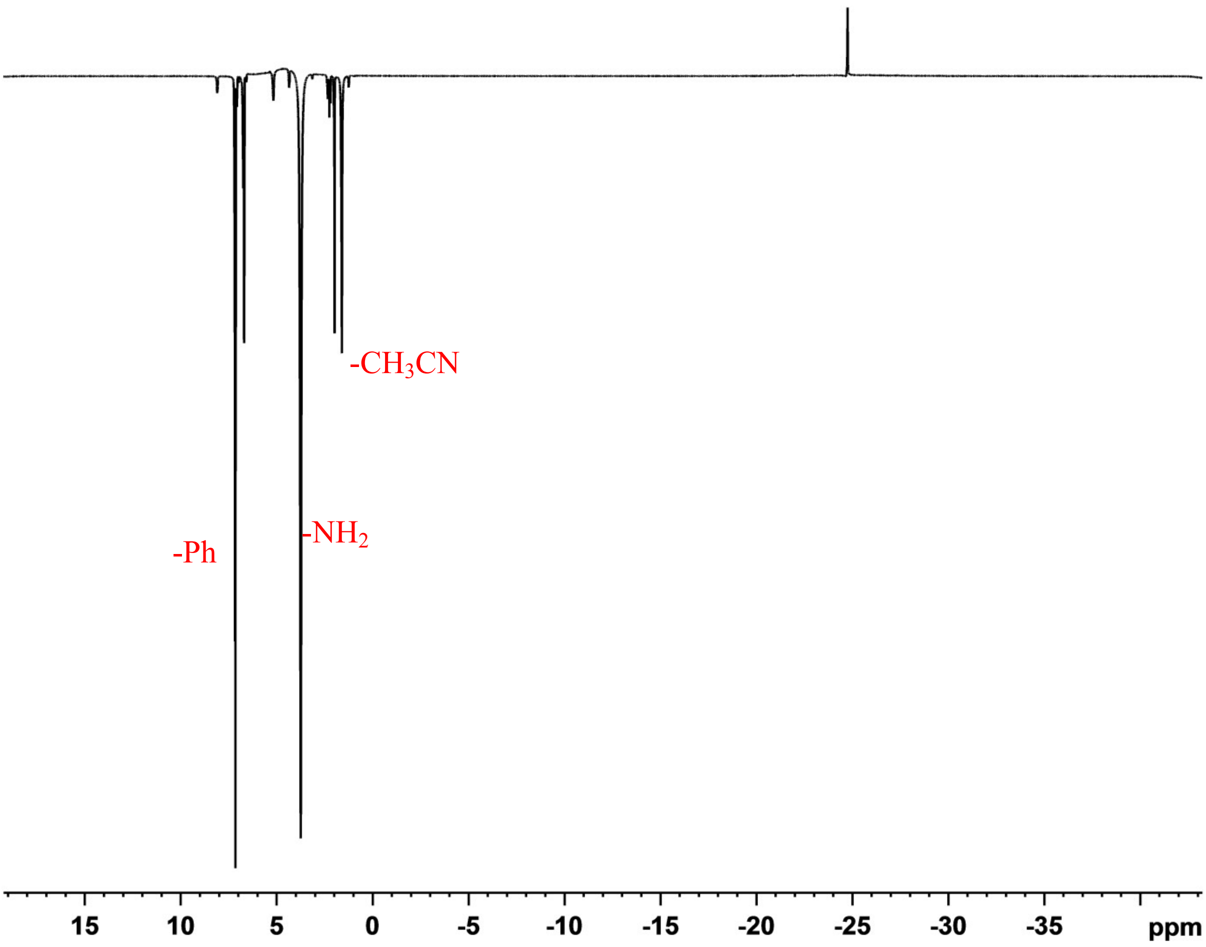

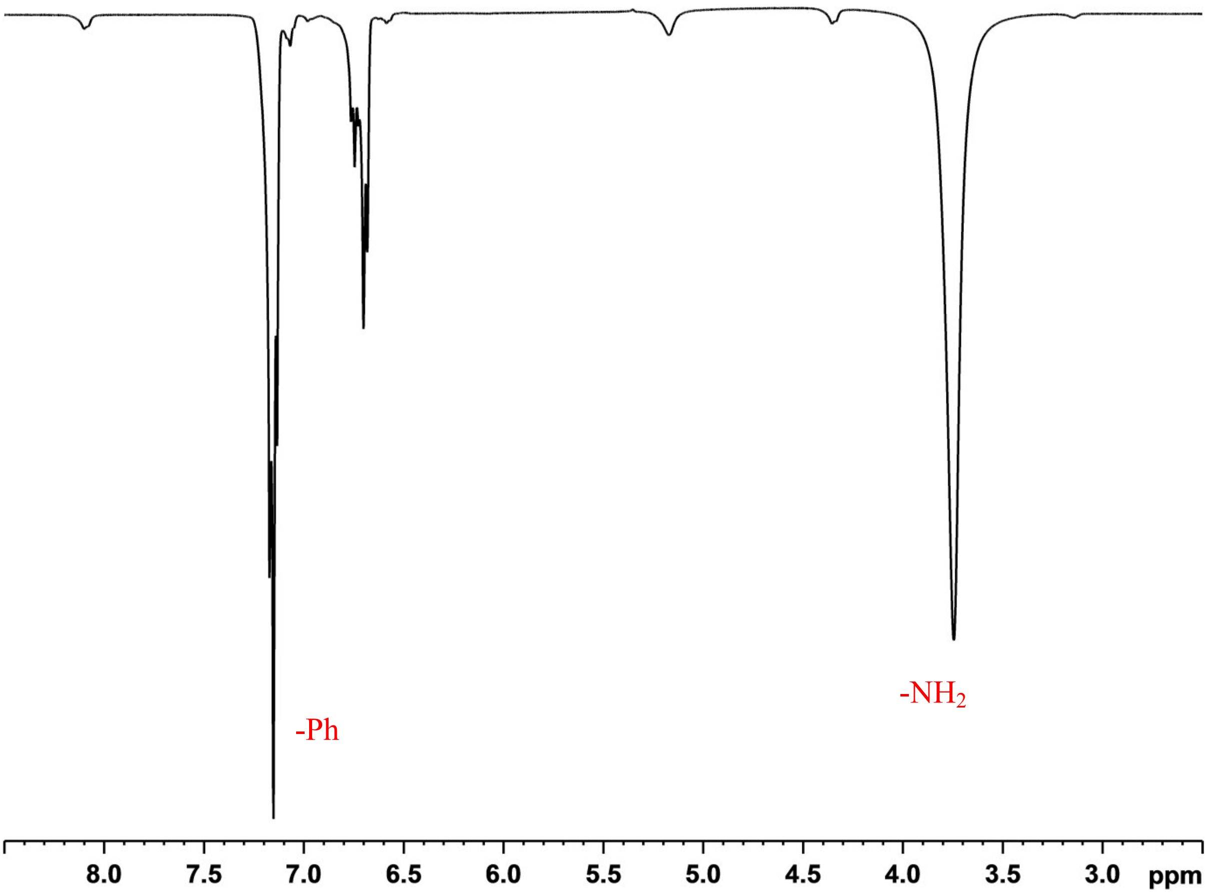

Fig. S10b ^{1}H SABRE enhanced NMR spectrum of aniline (top) and expansion (bottom)

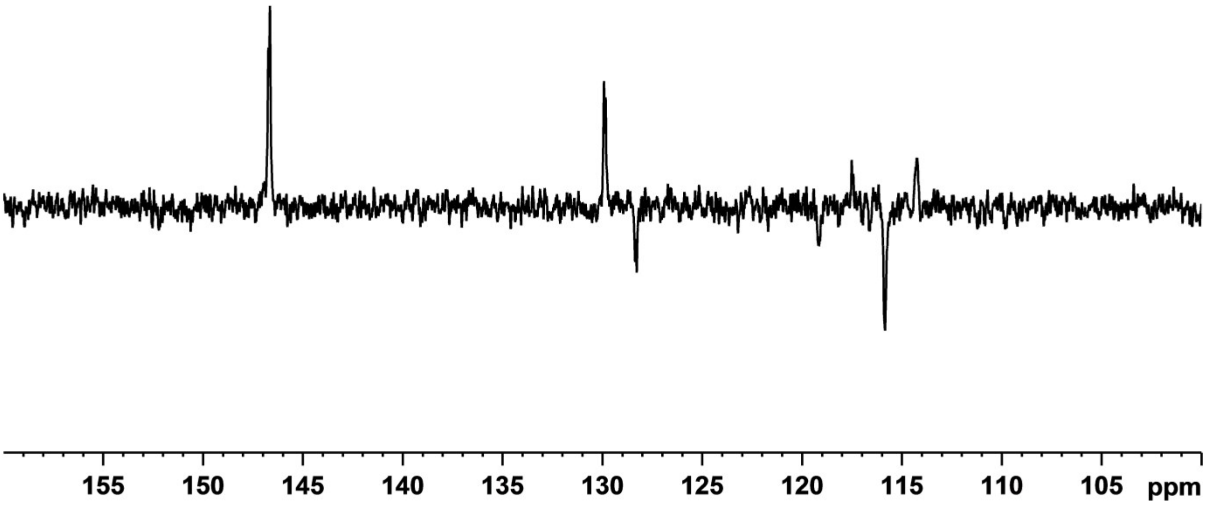

Fig. S10c ^{13}C SABRE enhanced NMR spectrum of spectrum for aniline

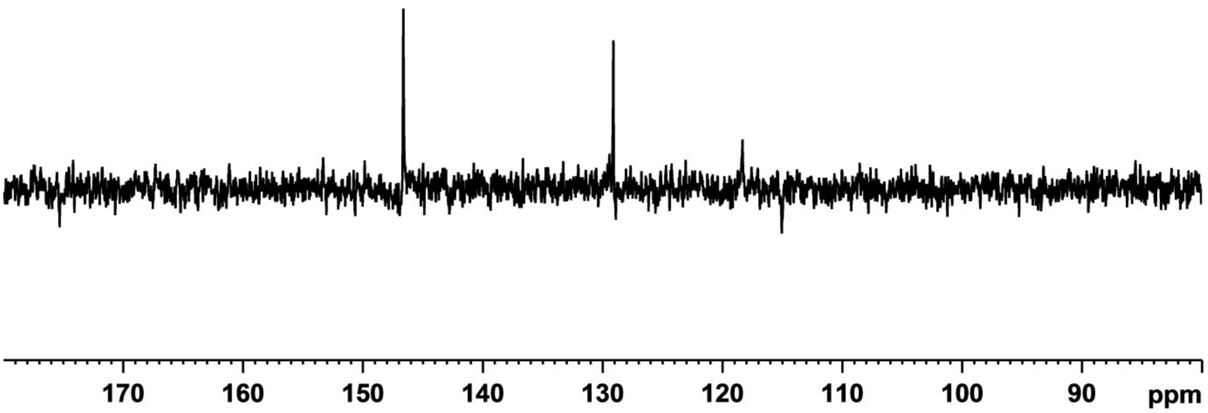

Fig. S10d INEPT ^{13}C SABRE enhanced NMR spectrum of phenoxyethylamine (J_{XH} = 120 Hz, B = 60 G)

4 Representative SABRE-RELAY NMR spectra and enhancement factors

- **Isopropylamine**

Fig. S11 reflects a series of single scan NMR spectra of isopropylamine: a) ^{1}H NMR spectrum thermally polarised x16, b) ^{1}H SABRE NMR spectrum and c) ^{13}C SABRE NMR spectrum using conditions: [IrCl(COD)(IMes)] (5 mM), isopropylamine (10 eq.), CD_2Cl_2 (0.6 mL); Substrate concentration: 50 mM. ^{1}H enhancement (fold/per proton): 220- (NH_2), 27-(CH) and 150-(CH_3)

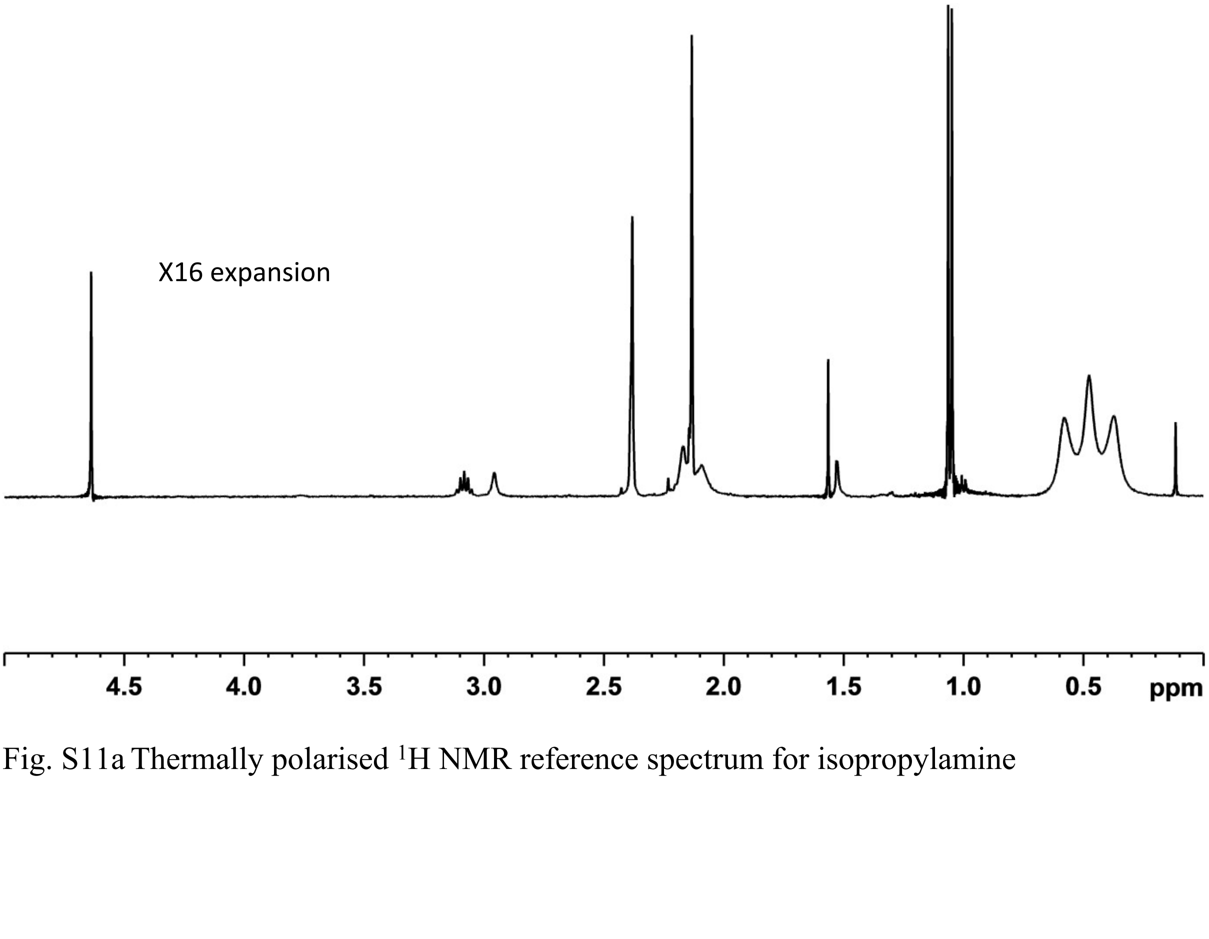

Fig. S11a Thermally polarised ^{1}H NMR reference spectrum for isopropylamine

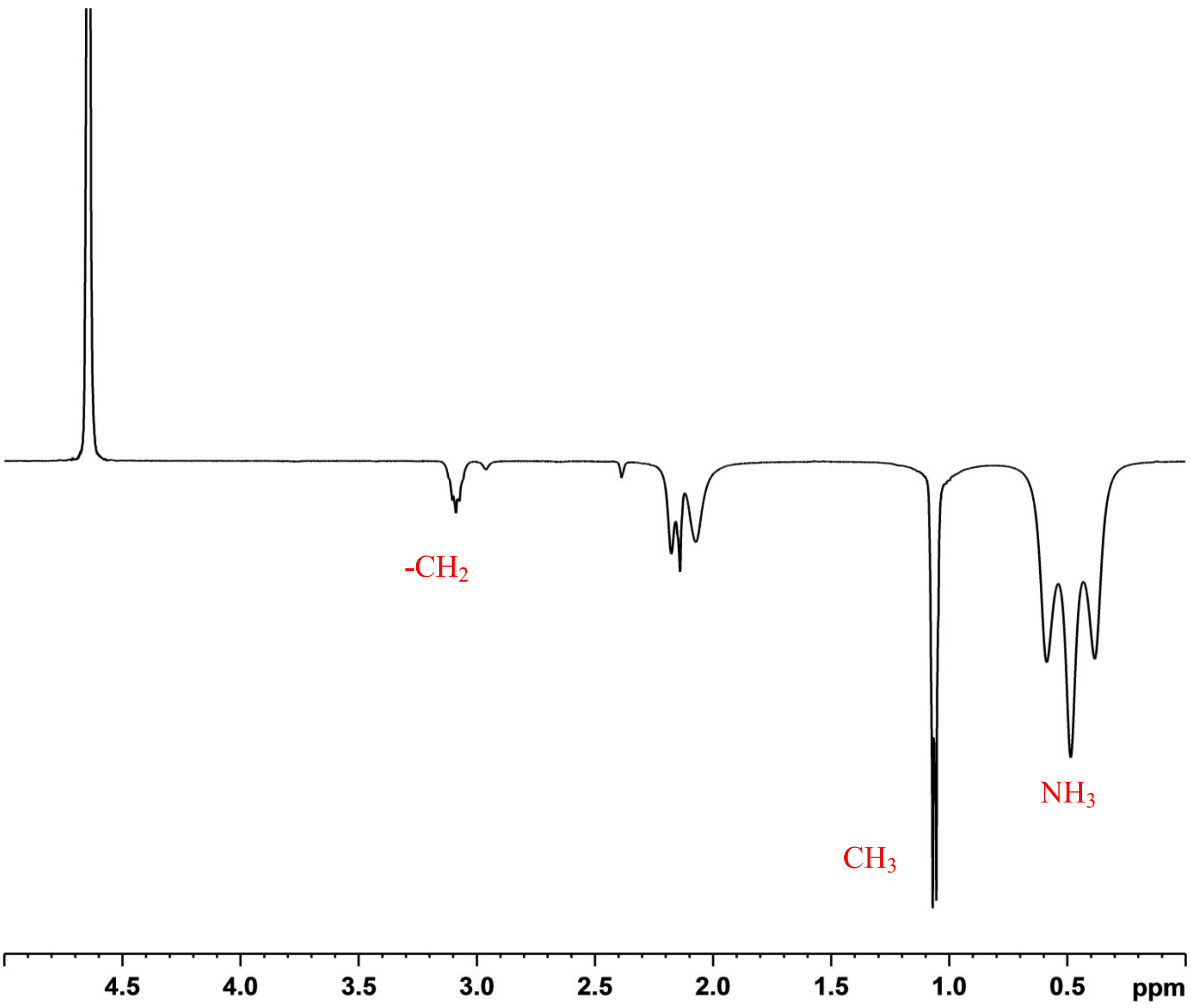

Fig. S11b ^{1}H SABRE enhanced NMR spectrum of isopropylamine (top) and expansion (bottom)

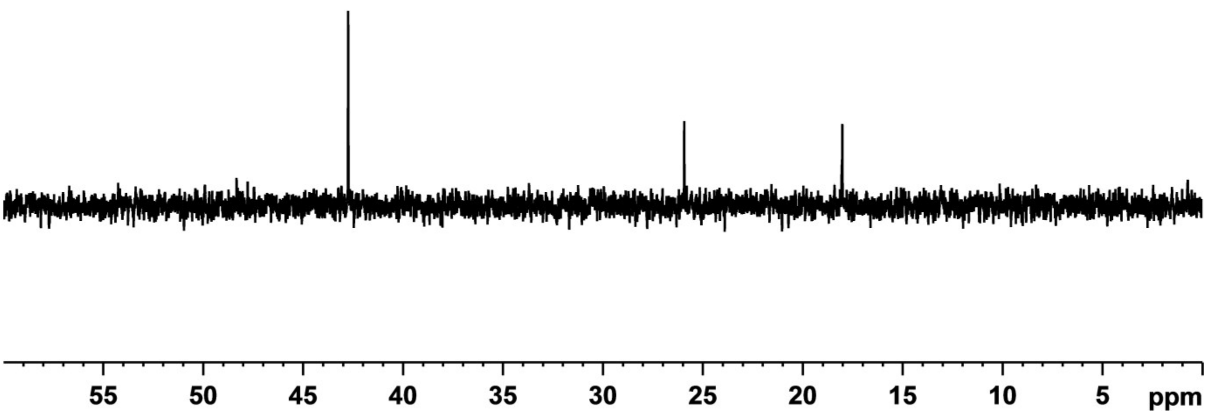

Fig. S11c INEPT ^{13}C SABRE enhanced NMR spectrum of isopropylamine (J_{XH} = 122 Hz, B = 60 G)

- **Dibenzylamine**

Fig. S12 reflects a series of single scan NMR spectra of dibenzylamineamine: a) ^{1}H NMR spectrum thermally polarised x64, b) ^{1}H SABRE NMR spectrum c) ^{13}C SABRE NMR spectrum and d) INEPT ^{13}C SABRE NMR spectrum using conditions: [IrCl(COD)(IMes)] (5 mM), dibenzylamine (10 eq.), CD_2Cl_2 (0.6 mL); Substrate concentration: 50 mM. ^{1}H enhancement (fold/per proton): 274-(NH-), 200-(CH_2) and 275-(Ph).

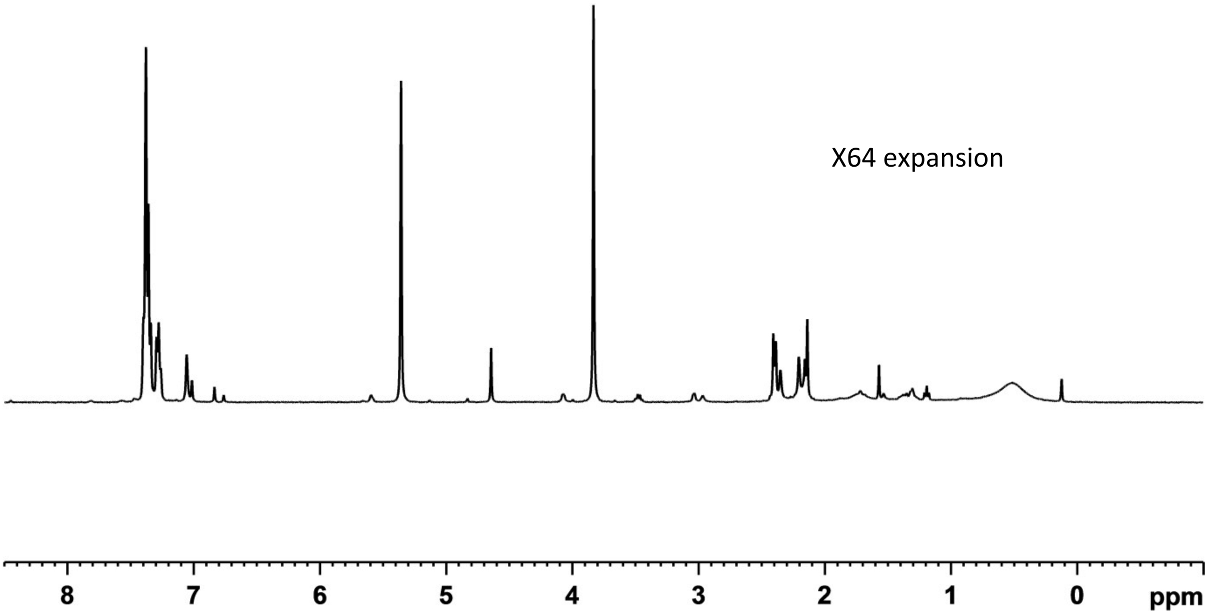

Fig. S12a Thermally polarised ^{1}H NMR reference spectrum of dibenzylamineamine

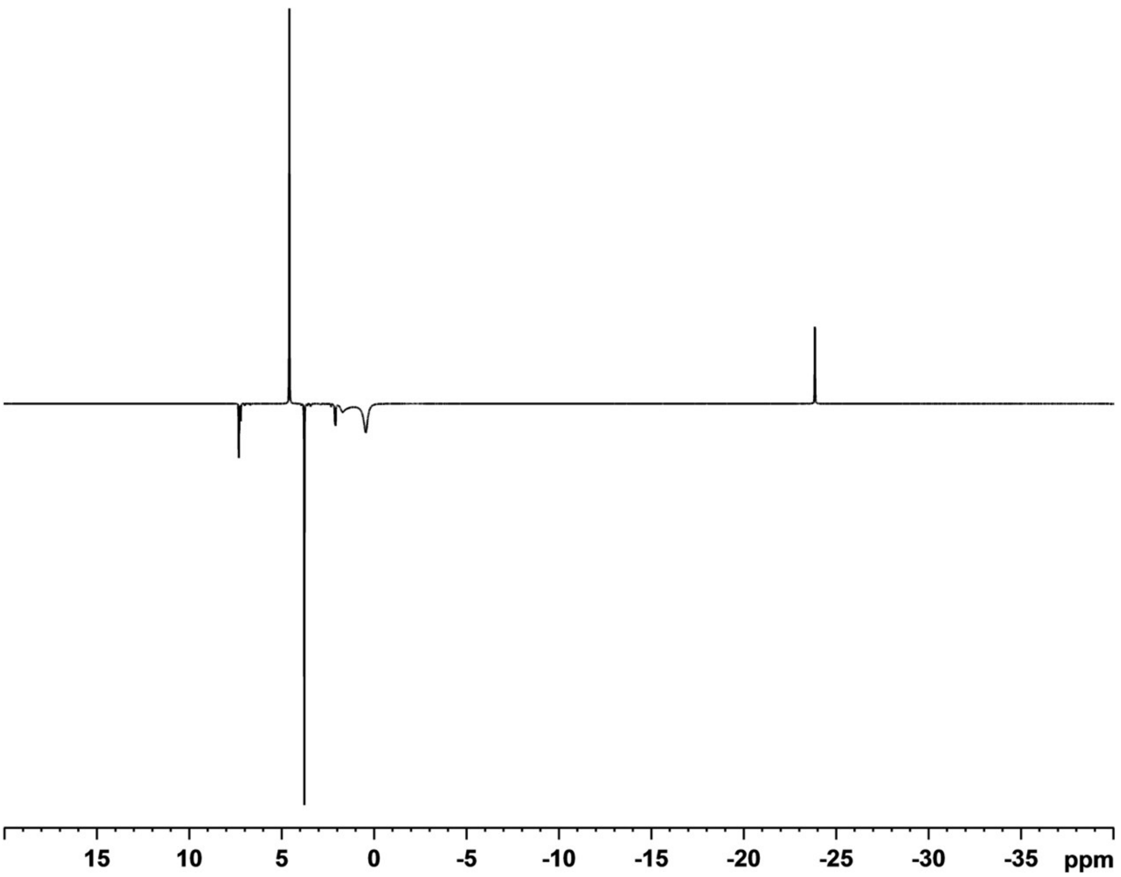

15
10
5
0
-5
-10
-15
-20
-25
-30
-35
ppm

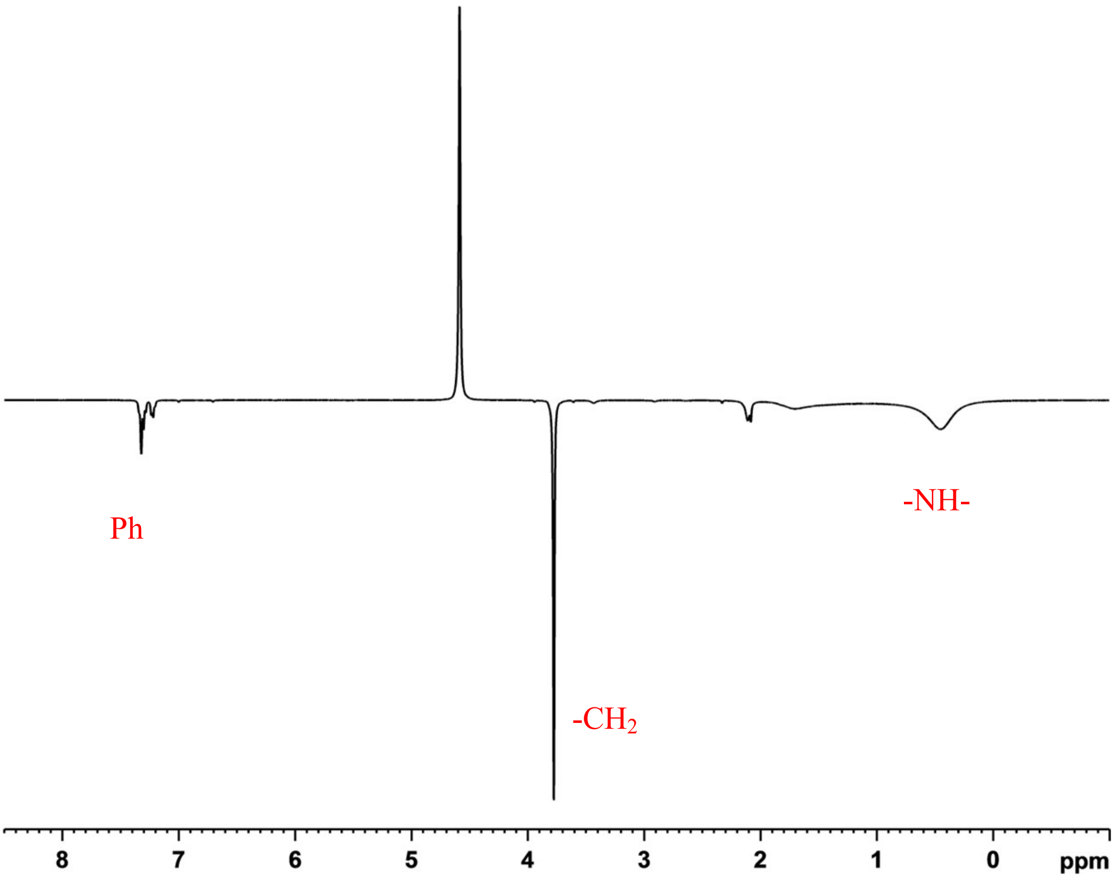

Ph
-NH-
-CH2
8
7
6
5
4
3
2
1
0
ppm

Fig. S12b ^{1}H SABRE enhanced NMR spectrum of dibenzylamineamine (top) and expansion (bottom)

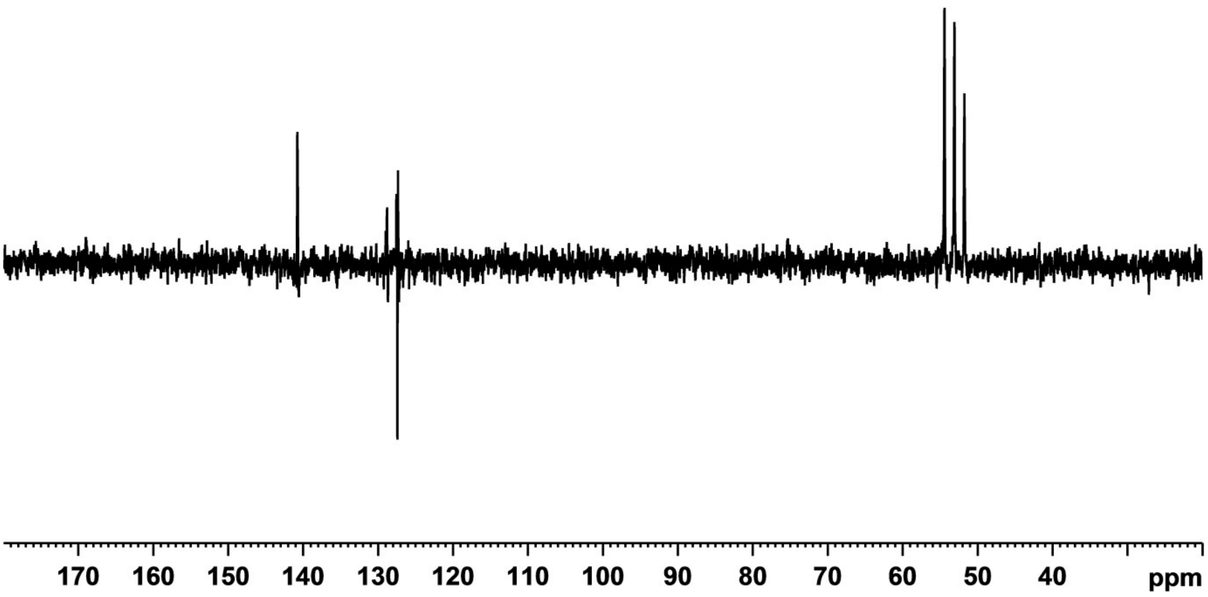

Fig. S12c ^{13}C SABRE enhanced NMR spectrum of dibenzylamineamine

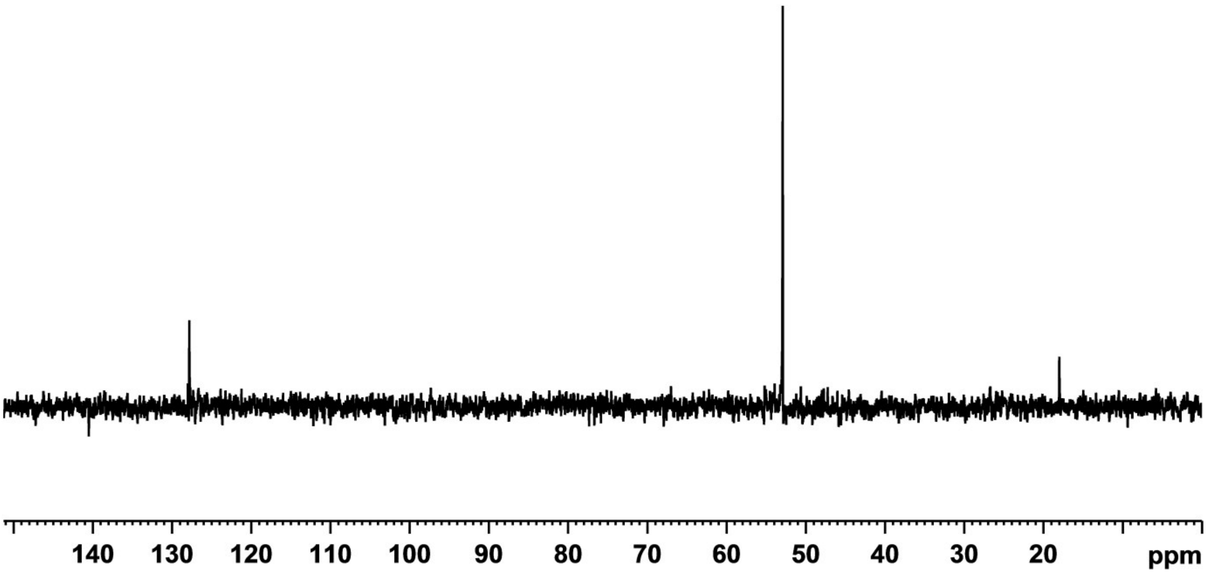

Fig. S12d INEPT ^{13}C SABRE enhanced NMR spectrum of dibenzylamineamine (J_{XH} = 122 Hz, B = 60 G)

- **Aniline**

Fig. S13 reflects a series of single scan NMR spectra of aniline: a) ^{1}H NMR spectrum thermally polarised x8 and b) ^{1}H SABRE NMR spectrum using conditions: [IrCl(COD)(IMes)] (5 mM), aniline (10 eq.), CD_2Cl_2 (0.6 mL); Substrate concentration: 50 mM. ^{1}H enhancement (fold/per proton): 150-(NH_2) and 9-(Ph)

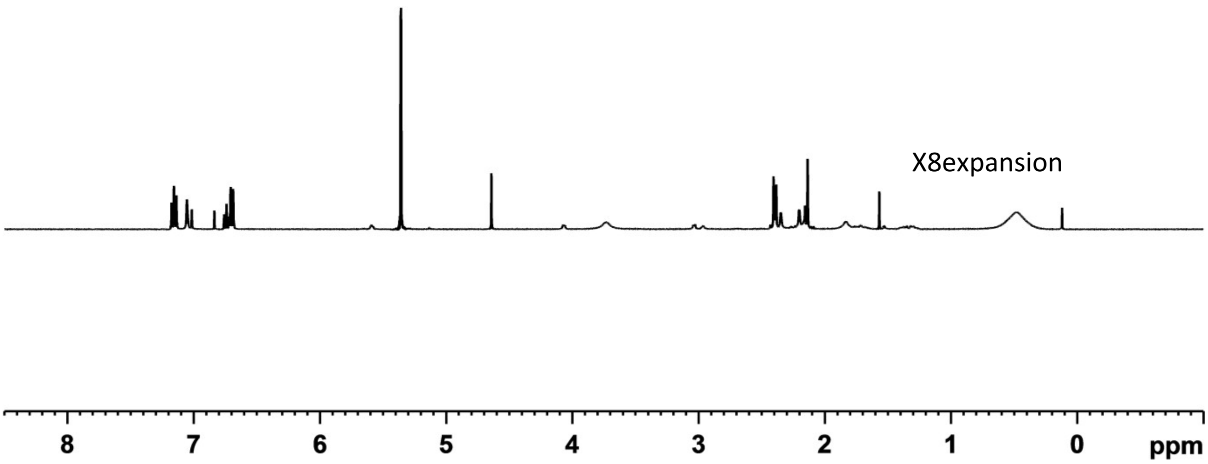

Fig. S13a Thermally polarised ^{1}H NMR reference spectrum of aniline

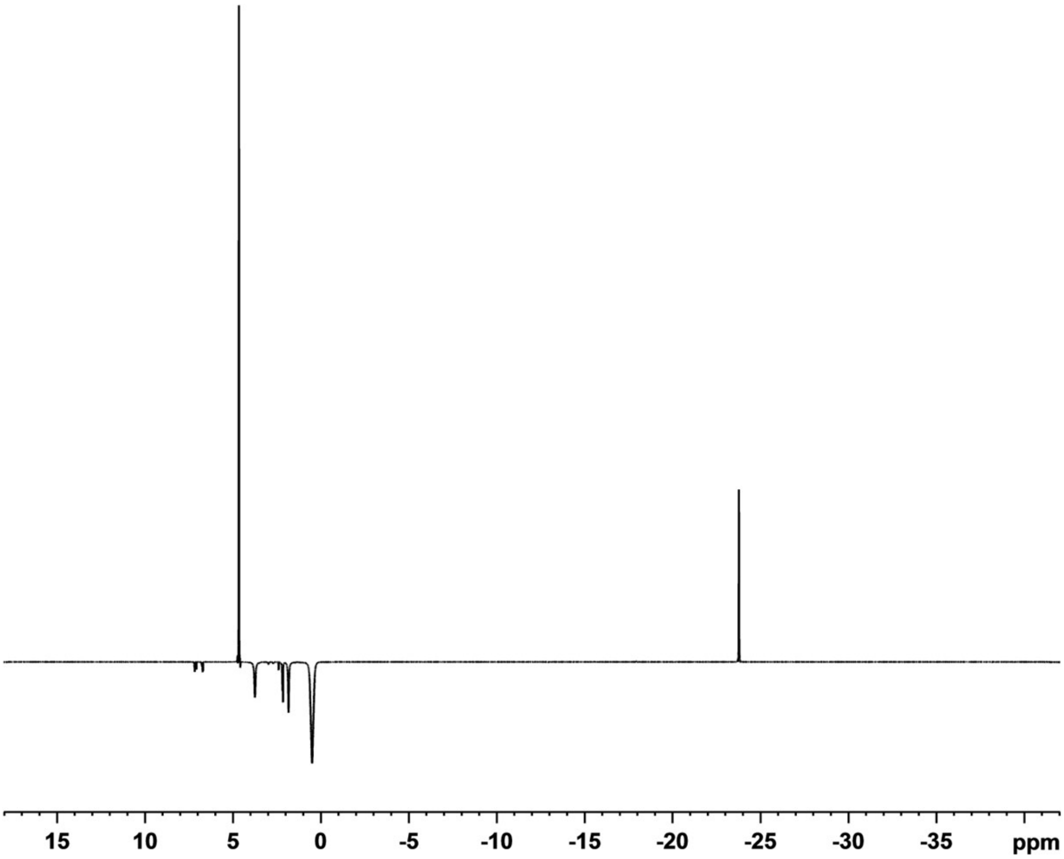

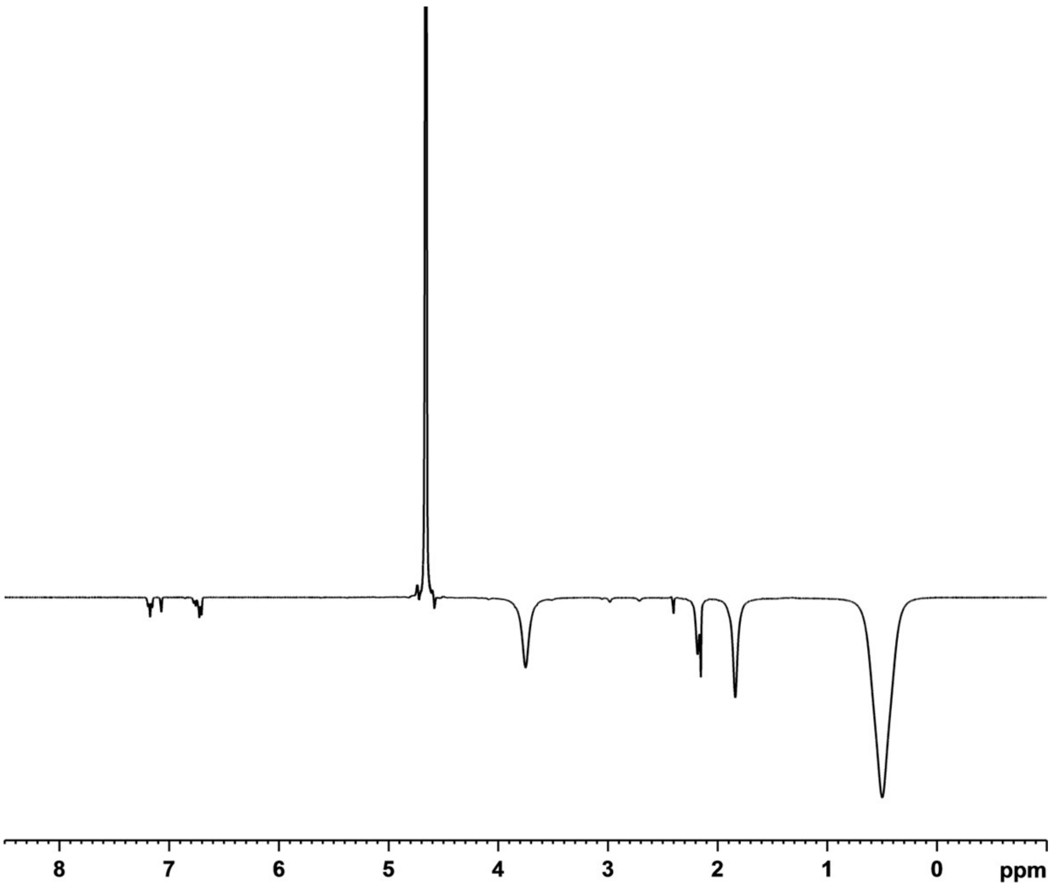

Fig. S13b ^{1}H SABRE enhanced NMR spectrum of aniline (top) and expansion (bottom)

5 Optimisation of the SABRE polarisation level of $BnNH_2$

5.1 Effect of varying the pressure of *p*-H_2

Increasing the pressure of *p*-H_2 was shown to improve the observed signal gains for the protons of $BnNH_2$ as shown in Figure S14.

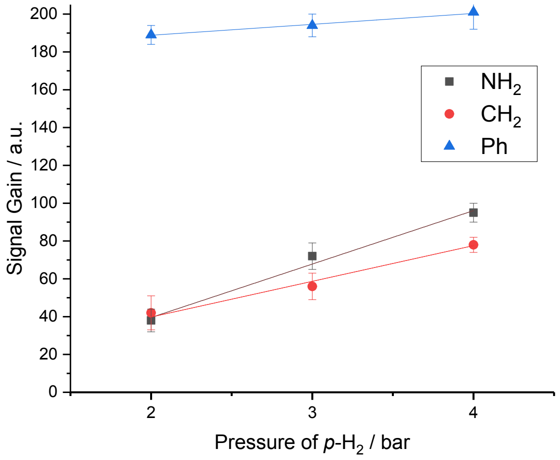

Fig. S14: Effect of p-H_2 pressure on the SABRE polarisation of $BnNH_2$ (10 eq.) using **1** (5 mM) in dichloromethane-d_2 solution

5.2 Effect of Temperature of SABRE catalysis

Increasing temperature at which the SABRE transfer occurs increased the observed signal gains of $BnNH_2$ as shown in Figure S15.

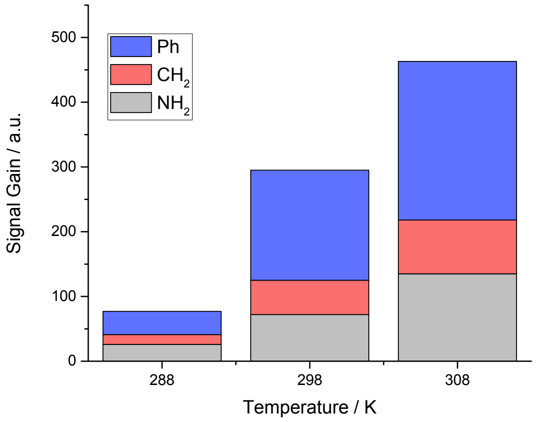

Fig. S15: Effect of temperature on the SABRE polarisation of $BnNH_2$ with **1** in dichloromethane-d_2 under 3-bar p-H_2 and transfer at 60 G.

6 Ligand exchange dynamics of 2-NH_3 and 2-$BnNH_2$

The kinetic behaviour of **2-NH_3** and **2-$BnNH_2$** under 3 bar H_2 was examined using well established exchange spectroscopy methods as follows:
A series of exchange spectroscopy (EXSY) measurements were performed to probe the dynamic behaviour of these systems. This process involved the selective excitation of a single resonance and the subsequent measurement of a ^1H NMR spectrum at time, t, after the initial pulse. The resulting measurements consisted of a series of data arrays such that t is varied

typically between 0.1 to 1.0 s, to encode the reaction profile. Integrals for the interchanging peaks in the associated ^{1}H EXSY spectra were obtained and converted into a percentage of the total detected signal. These data were then analysed as a function of the mixing time according to a differential kinetic model.

Ligand	Temperature / K	Rate of Dissociation / s^{-1}
**Equatorial NH_3**	298	0.32 ± 0.01
**Hydride**	298	1.65 ± 0.02
**Equatorial NH_3**	308	10.42 ± 0.06
**Hydride**	308	1.41 ± 0.01

Table S1: Observed rate of ligand loss from **2-NH_3** at varying temperatures.

Ligand	Temperature / K	Rate of Dissociation / s^{-1}
**Equatorial NH_3**	298	3.33 ± 0.03
**Hydride**	298	2.84 ± 0.05
**Equatorial NH_3**	308	9.85 ± 0.04

Table S2: Observed rate of ligand loss from **2-$BnNH_2$** at the indicated temperature.

7 NMR Characterisation data for the active SABRE catalysts featuring in this paper

7.1 NMR characterisation data for 2-NH_3

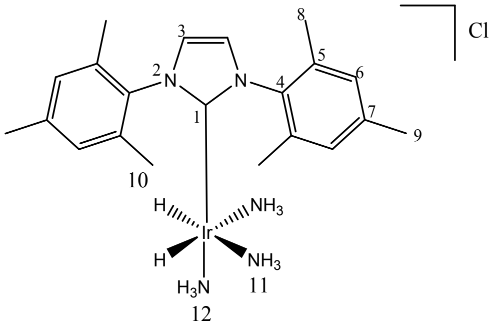

Resonance number	^{1}H (ppm)	^{13}C (ppm)	^{15}N (ppm)
**1**		153.8	
**2**			190.83
**3**	6.80	121.5	
**4**		138.11	
**5**		135.25	
**6**	7.00	129.00	
**7**		138.64	
**8**	2.10	18.30	
**9**	2.35	20.78	
**10**	-23.61		
**11**	2.21 (br, s, J_{NH} = 67 Hz)		-34.9
**12**	2.84 (br, s, J_{NH} = 69 Hz)		-46.5

7.2 NMR characterisation data for 2-$BnNH_2$

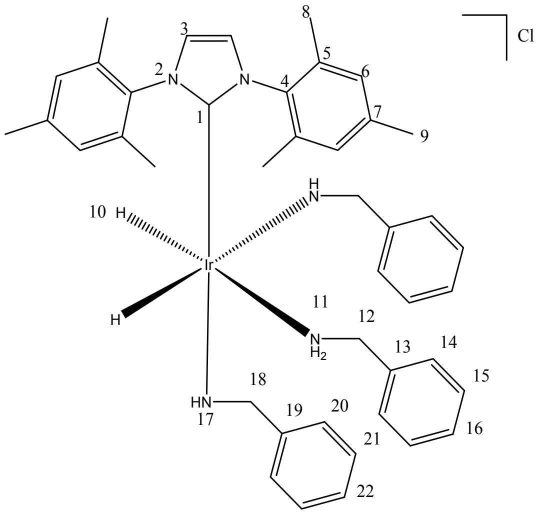

Resonance number	^{1}H (ppm)	^{13}C (ppm)	^{15}N (ppm)
**1**		153.8	
**2**			192.83
**3**	6.80	121.73	
**4**		138.11	
**5**		135.25	
**6**	6.90	129.29	
**7**		138.64	
**8**	2.17	18.30	
**9**	2.21	20.78	
**10**	-23.95		
**11**	5.00 (br, dt, J_{HH} = 5 and 11 Hz, 2H, J_{15NH} = 68 Hz)<br>2.30 (br, t, J_{HH} = 11 Hz, 2H, J_{15NH} = 68 Hz)		-6.4
**12**	3.65 (dt, J_{HH} = 5 and 15 Hz, 2 H), 3.30 (ddd, J_{HH} = 3, 12 and 15 Hz, 2H)	53.14	
**13**		141.8	
**14**	7.35 (d, J_{HH} = 5 Hz)	128.33	
**15**	7.25 (t, J_{HH} = 5 Hz)	126.88	
**16**	~7.28 - overlap	-	
**17**	4.25 (t, J_{HH} = 7 Hz, 2H, J_{15NH} = 69 Hz)		-13.4
**18**	3.85 (t, J_{HH} = 7 Hz, 2H)	57.26	
**19**		-	
**20**	7.30	128.35	
**21**	~7.28 - overlap	-	
**22**	~7.28 - overlap	-	

7.3 NMR characterisation data for 2-PEA

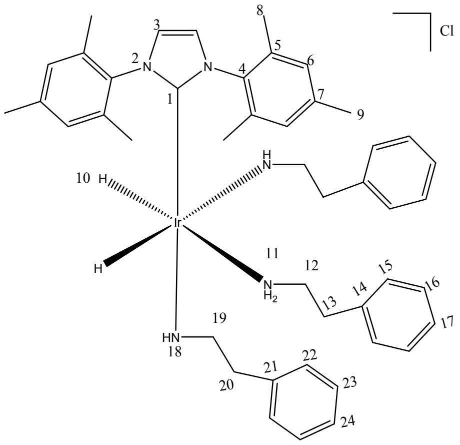

Resonance number	^{1}H (ppm)	^{13}C (ppm)	^{15}N (ppm)
**1**		153.8	
**2**			192.21
**3**	6.75	121.73	
**4**		138.14	
**5**		135.06	
**6**	7.0	129.4	
**7**		138.64	
**8**	2.17	18.30	
**9**	2.21	21.10	
**10**	-23.95		
**11**	4.42.00 (t, J_{HH} = 11 Hz, 2H, J_{15NH} = 68 Hz)<br>2.11 (br, t, J_{HH} = 11 Hz, 2H, J_{15NH} = 68 Hz)		-7.2
**12**	2.41 (dt, J_{HH} = 9 and 11 Hz, 2 H), 3.30 (2H, overlap)	50.7	
**13**			
**14**		141.8	
**15**	7.32 (dd, J_{HH} = 7 Hz)	128.33	
**16**	7.25 (t, J_{HH} = 7 Hz)	126.88	
**17**	~7.28 - overlap	-	
**18**	3.685 (t, J_{HH} = 5.9 Hz, 2H, J_{15NH} = 69 Hz)		-16.2
**19**	2.78 (t, J_{HH} = 6.15 Hz, 2H)	53.9	
**20**		-	
**21**	7.32	128.33	
**22**	~7.2 - overlap	-	
**23**	~7.12 - overlap	-	

7.4 NMR characterisation data for $[Ir(H)_2(IMes)(aniline)_2(MeCN)]Cl$

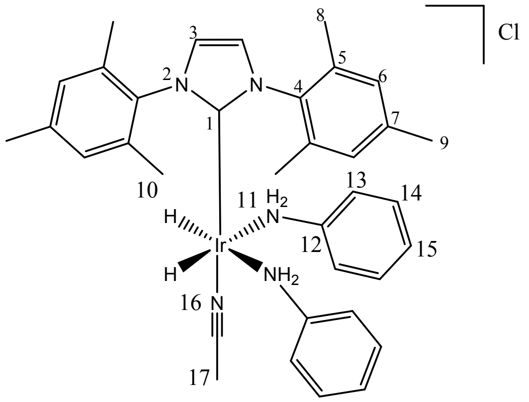

Resonance number	^{1}H (ppm)	^{13}C (ppm)	^{15}N (ppm)
**1**		153.8	
**2**			193.75
**3**	6.90	121.5	
**4**		138.11	
**5**		135.25	
**6**	7.00	129.00	
**7**		138.64	
**8**	2.10	18.30	
**9**	2.35	20.78	
**10**	-24.72	-	-
**11**	4.34 (d, J_{HH} = 8 Hz), 8.00 (d, J_{HH} = 8 Hz)	-	-25
**12**			
**13**	6.50 (d, J_{HH} = 7 Hz)		
**14**	8.27 (t, J_{HH} = 7 Hz)		
**15**	6.78 (d, J_{HH} = 7 Hz)		
**16**			176
**17**	0.95 (s)		
